# Genetic and Virulence Characteristics of a Hybrid Atypical Enteropathogenic and Uropathogenic *Escherichia coli* (aEPEC/UPEC) Strain

**DOI:** 10.3389/fcimb.2020.00492

**Published:** 2020-09-29

**Authors:** Tiago B. Valiatti, Fernanda F. Santos, Ana C. M. Santos, Júllia A. S. Nascimento, Rosa M. Silva, Eneas Carvalho, Rita Sinigaglia, Tânia A. T. Gomes

**Affiliations:** ^1^Departamento de Microbiologia, Imunologia e Parasitologia, Escola Paulista de Medicina, Universidade Federal de São Paulo, São Paulo, Brazil; ^2^Laboratório de Bacteriologia, Instituto Butantan, São Paulo, Brazil; ^3^Centro de Microscopia Eletrônica, Escola Paulista de Medicina, Universidade Federal de São Paulo, São Paulo, Brazil

**Keywords:** *Escherichia coli*, UPEC, hybrid pathogenic strain, virulence factor, ExPEC, urinary tract infection, atypical EPEC

## Abstract

Hybrid strains of *Escherichia coli* combine virulence traits of diarrheagenic (DEC) and extraintestinal pathogenic *E. coli* (ExPEC), but it is poorly understood whether these combined features improve the virulence potential of such strains. We have previously identified a uropathogenic *E. coli* (UPEC) strain (UPEC 252) harboring the *eae* gene that encodes the adhesin intimin and is located in the locus of enterocyte effacement (LEE) pathogenicity island. The LEE-encoded proteins allow enteropathogenic *E. coli* (EPEC) and enterohemorrhagic *E. coli* (EHEC) to form attaching and effacing (A/E) lesions in enterocytes. We sought to characterize UPEC 252 through whole-genome sequencing and phenotypic virulence assays. Genome analysis unveiled that this strain harbors a complete LEE region, with more than 97% of identity comparing to E2348/69 (EPEC) and O157:H7 Sakai (EHEC) prototype strains, which was functional, since UPEC 252 expressed the LEE-encoded proteins EspB and intimin and induced actin accumulation foci in HeLa cells. Phylogenetic analysis performed comparing 1,000 single-copy shared genes clustered UPEC 252 with atypical EPEC strains that belong to the sequence type 10, phylogroup A. Additionally, UPEC 252 was resistant to the bactericidal power of human serum and colonized cells of the urinary (T24 and HEK293-T) and intestinal (Caco-2 and LS174T) tracts. Our findings suggest that UPEC 252 is an atypical EPEC strain that emerges as a hybrid strain (aEPEC/UPEC), which could colonize new niches and potentially cause intestinal and extraintestinal infections.

## Introduction

Urinary tract infection (UTI) is one of the most prevalent diseases worldwide, affecting both community and hospitalized subjects, resulting in impairment of the patients' life quality (Terpstra and Geerlings, 2016; Wurpel et al., 2016). Several factors, such as surgical manipulation, diabetes, previous hospitalizations, and catheterization, are related to a higher risk for developing this disease (Saltoglu et al., 2015; Redder et al., 2016). UTI most often results from bacteria transitorily present in the gut microbiota that colonize the perineum and ascend through the urethra. Alternatively, UTI may occur after the introduction of bacteria directly into the urinary tract, during the use of a bladder catheter or certain sexual practices (Donnenberg and Welch, 1996; Finer and Landau, 2004). Characteristics of the host, especially those associated with immunity, also contribute to the occurrence and severity of infections in the urinary tract.

The main agents of UTI are Gram-negative bacilli of the family *Enterobacteriaceae*. Among them, uropathogenic *Escherichia coli* (UPEC) are the most frequent (Donnenberg and Welch, 1996; Heilberg and Schor, 2003), responding for about 80% of all cases worldwide (Ronald, 2003; Hooton, 2012; Korbel et al., 2017). UPEC strains belong to a group of *E. coli* clones that are collectively referred to as extraintestinal pathogenic *E. coli* (ExPEC), which are adapted to colonize and cause disease in different extraintestinal host sites (Russo and Johnson, 2000). The ability of ExPEC strains to colonize and develop extraintestinal diseases is due to the presence of a variety of virulence factors, including adhesins, toxins, capsules, invasins, and iron uptake systems that are encoded in Pathogenicity islands (PAI) present in chromosomes or plasmids (Dale and Woodford, 2015). Currently, more than 50 virulence factors have their role in ExPEC pathogenicity established (Johnson and Russo, 2018), but the ability of ExPEC to colonize a variety of host sites varies among strains reflecting their genetic diversity.

Despite the number of virulence factors related to ExPEC pathogenicity, its classification is primarily performed by the isolation site, and their pathotypes defined by the infection source, with uropathogenic *E. coli* (UPEC) being the most prevalent ExPEC pathotype. Many studies focus on UPEC virulence and epidemiology, mainly because of the disease's burden. Johnson et al. (2003) described a set of virulence factors that could identify ExPEC strains by their intrinsic virulence, and are thus considered more virulent strains (presence of at least two among five markers: *pap, sfa/foc, afa/dra, iuc/iut, kpsMTII*). Later, Spurbeck et al. (2012) described another set of virulence markers that are epidemiologically linked with the uropathogenic potential (simultaneous presence of *chuA, fyuA, vat*, and *yfcV*). Together these methodologies enable the screening of the ExPEC strains that either harbor intrinsic virulence or uropathogenic potential, in diverse sources, like water, food, and gut, favoring the identification of their reservoirs. However, these criteria fail to classify all strains isolated from extraintestinal infections (Spurbeck et al., 2012; Santos et al., 2013, 2020b; Freire et al., 2020) and some clinical lineages with epidemiological importance also do not meet these criteria (Bert et al., 2010; Olesen et al., 2012; Manges and Johnson, 2015; Manges et al., 2017, 2019; Campos et al., 2018; Yamaji et al., 2018; Santos et al., 2020a,b).

Other clones of *E. coli* can cause intestinal infections by different mechanisms and are collectively referred to as diarrheagenic *E. coli* (DEC). DEC strains are classified into six pathotypes named enterotoxigenic *E. coli* (ETEC), enteroinvasive *E. coli* (EIEC), enteroaggregative *E. coli* (EAEC), Shiga toxin-producing *E. coli* (STEC), diffusely adherent *E. coli* (DAEC), and enteropathogenic *E. coli* (EPEC) (Kaper and Nataro, 1998; Croxen et al., 2013; Gomes et al., 2016). EPEC strains are divided into typical (tEPEC) and atypical (aEPEC) based on the presence of the EAF (EPEC adherence factor) plasmid (pEAF) in tEPEC and its absence in aEPEC (Trabulsi et al., 2002). pEAF encodes a bundle forming pilus (BFP) that promotes bacterial aggregation and, consequently, the formation of compact microcolonies that characterize a localized adhesion (LA) pattern on the surface of HeLa and HEp-2 epithelial cells (Scaletsky et al., 1984; Girón et al., 1991).

Reports of *E. coli* strains that caused intestinal and extraintestinal infections in the same host are uncommon. However, recent studies have identified ExPEC strains promoting intestinal infections followed by bloodstream infections (Mariani-Kurkdjian et al., 2014; Kessler et al., 2015). These strains that contain virulence factors of intestinal and extraintestinal pathotypes are considered hybrid and potentially more virulent pathogens. In these studies, the horizontal gene transfer (HGT) of virulence factors occurred from ExPEC to DEC and DEC to ExPEC.

Despite the recognized potential of HGT between pathogenic *E. coli* strains, only a few studies have described this phenomenon among DEC and UPEC in strains isolated from UTI and in these studies the genetic background of strains involved was not evaluated (Olesen et al., 1994; Garcia et al., 2000; Keller et al., 2002; Matar et al., 2005; Ogura et al., 2007; Wallace-Gadsden et al., 2007; Abe et al., 2008; Lara et al., 2017; Santos et al., 2020a). A study evaluating fecal EAEC isolates identified that some strains produced alpha-hemolysin (*hlyA*) and P fimbria (*pap*) (Suzart et al., 1999), characteristics often found in UPEC strains, suggesting that certain EAEC strains with specific virulence genes would be potentially uropathogenic. Assessing the virulence factors of strains isolated from UTI, we have previously (Falsetti, 1998; Abe et al., 2008) demonstrated in some UPEC isolates the presence of the *aggR* and *aat* genes, as well as the aggregative adhesion (AA) pattern on HeLa cells, which are properties that define the EAEC pathotype. In addition to the UPEC isolates bearing EAEC virulence genes, in the referred study, we identified a UPEC strain (UPEC 252) carrying the *eae* gene (Abe et al., 2008), which encodes intimin, an outer membrane adhesive protein that is fundamental for the intimate adhesion of EPEC and EHEC strains to intestinal cells and subsequent formation of attaching/effacing (A/E) lesions (Kaper et al., 2004). A/E lesions are characterized by intimate bacterial adhesion to the host cells, which undergoes the eradication of microvilli and accumulation of actin filaments underneath the adhered bacteria, thus promoting the formation of pedestal-like structures on the cell surface (Moon et al., 1983). This type of lesion can be identified in non-intestinal (HeLa and HEp-2 cells) and intestinal epithelial (Caco-2) cell lineages *in vitro*, as well as in animal and human enterocytes *in vivo* (Kaper et al., 2004). The genes involved in A/E lesion formation are contained in a chromosomal PAI called the locus of enterocyte effacement (LEE). Besides *eae*, LEE encodes a type III secretion system (T3SS) and several effectors and regulatory proteins, whose activities culminate in the production of the lesion (Mcdaniel et al., 1995). Regardless of the detection of the *eae* gene in UPEC 252, the genetic background of the strain has not been properly evaluated, and its relationship with other pathogenic *E. coli* is unknown. Furthermore, it is not known whether the DEC virulence genes found in this strain are complete, nor if they are expressed and play a role in the bacterial interaction with intestinal and urinary tract cells. Therefore, this study aimed to provide information about the genetic and virulence properties of UPEC 252, an *E. coli* that was isolated from UTI and carries the *eae* gene characteristic of EPEC and EHEC.

## Materials and Methods

### Bacterial Strain

UPEC 252 was isolated, in 1998, from a 1-month-old female patient hospitalized at Hospital São Paulo, São Paulo, Brazil, with urinary tract infection (Falsetti, 1998; Abe et al., 2008). The strain was stored in Lysogeny broth (LB) supplemented with 15% glycerol and kept at −80°C. For routine use, it was grown in LB at 37°C for ~18 h.

### Genome Sequencing, Assembly, Annotation, and Data Analysis

The genome of UPEC 252 was sequenced as previously published (Valiatti et al., 2019). Briefly, DNA was extracted using the Wizard® Genomic DNA Purification Kit and sequenced on an Illumina Hiseq1500 platform. The paired-end reads were assembled *de novo* using SPAdes (version 3.12.0) (Bankevich et al., 2012) and the annotation was performed using Prokka version 1.13.3 (Seemann, 2014). The reads used for UPEC 252 genome assembly were deposited in the Sequence Read Archive (SRA) at NCBI under the accession number SRR9317828, and the whole-genome sequences (WGS) were deposited in the GenBank database under the accession number VFST00000000 (Valiatti et al., 2019).

The WGS of UPEC 252 was submitted to the Center for Genomic Epidemiology (CGE) to determine the strain sequencing type (Multi-Locus Sequence Typing - MLST version 2.0) (Larsen et al., 2012), virulence profile (VirulenceFinder 2.0) (Joensen et al., 2014), resistance profile (ResFinder 3.1) (Zankari et al., 2012), and serotype (SerotypeFinder version 1.1) (Joensen et al., 2015). To analyze the presence of virulence and resistance genes, we also used ecoli_VF collection v0.1 (Leimbach, 2016) and the ABRicate program^1^, which employes multiple databases: VFDB (Chen et al., 2016), ARG-ANNOT (Gupta et al., 2014), CARD (Jia et al., 2017), PlasmidFinder (Carattoli et al., 2014), EcOH (Ingle et al., 2016), MEGARes 2.0 (Doster et al., 2020), and NCBI AMRFinderPlus (Feldgarden et al., 2019). The phylogenetic typing of the strain was determined using the ClermonTyping tool (Beghain et al., 2018). Confirmation of the identified genes of the LEE region and the non-LEE encoded genes was carried out by aligning the DNA sequences of UPEC 252 with the DNA sequences deposited at the National Center for Biotechnological Information (NCBI) using the BLAST program.

### Comparative Genome Analyses of UPEC 252

To identify the UPEC 252 genome rearrangement, gain and loss, different strategies were used. First, the annotated genome of UPEC 252 was aligned using progressiveMAUVE (Darling et al., 2010). In another approach, pangenome analysis was performed using the Roary pipeline (Page et al., 2015). In both methods, the genome of UPEC 252 was compared with the genome of the following strains: prototype EPEC E2348/69 (GCF_000026545.1), aEPEC 4581-2 (QYYF00000000.1), prototype EHEC O157:H7 Sakai (GCF_000008865.2), EHEC O111:H- 11128 (GCF_000010765.1), prototype UPEC CFT073 (GCF_000007445.1), prototype UPEC 536 (GCF_000013305.1), prototype UPEC UMN026 (GCF_000026325.1), an isolate from asymptomatic bacteriuria VR50 (GCF_000968515.1), and K12 derived MG1655 (GCF_000005845.2).

### UPEC 252 Phylogenetic Analysis

The phylogenetic tree that includes UPEC 252 was built using the Phylogenetic tree tool of the Pathosystems Resource Integration Center (PATRIC) (Wattam et al., 2017). A total of 140 *E. coli* public genomes were used to build the tree, 95 genomes of which were randomly chosen among strains belonging to ST10 (Supplementary Table 1), while 45 others were selected by similarity using the Mash algorithm in the PATRIC similarity genome service (Supplementary Table 1). The tree was built applying the codon tree methodology employing the alignment of 1,000 single-copy coding-genes in the RAxML matrix. The *E. coli* strains UMN026 (ST597-D), CFT073 (ST73-B2), 536 (ST127-B2), S88 (ST95-B2), E2348/69 (ST15-B2), O157:H7 Sakai (ST11-E), and O111:H- 11128 (ST16-B1) were added to the phylogenetic tree as outgroups as well as the *Escherichia fergusonni* strain ATCC35469. The final layout and annotation were accomplished using iTOL v.4. (Letunic and Bork, 2019).

### Analysis of Intimin Production and EspB Secretion by Immunoblotting

For the immunoblotting assays, the bacterial inoculum was standardized sub-culturing the strains at a dilution of 1/50 into DMEM until they reached an OD600 ≅1.

Intimin production was analyzed using total protein extract, followed by immunoblotting, as previously described by Towbin et al. (1979) using a monospecific polyclonal antibody against the conserved region of this protein (Int388-667) (Menezes et al., 2010).

Secreted proteins were extracted as described by Zarivach et al. (2007), and an immunoblotting assay using an anti-EspB primary antibody (Guirro et al., 2013) and anti-rabbit secondary IgG antibody (whole molecule)-Peroxidase (Sigma, Saint Louis, EUA) was employed to evaluate the ability of UPEC 252 to produce T3SS. A protein extract from the typical EPEC prototype strain E2348/69 was used as a positive control (Santos et al., 2019).

### Cell Culture and Maintenance

HeLa (ATCC® CCL-2), HEK293T (ATCC® CRL-1573), Caco-2 (ATCC® HTB-37TM), and LS174T (ATCC® CL-188) cell lineages were cultivated using Dulbecco's Modified Eagle Medium (DMEM), high glucose, GlutaMax™ (Gibco-ThermoFisher Scientific, USA) (2 g/L sodium bicarbonate, 4.5 g/L glucose) containing 15 mM HEPES (Sigma, Saint Louis, USA), 1x Penicillin-Streptomycin-Neomycin (PSN) antibiotic mixture (GiBCO, USA), and 10% bovine fetal serum (BFS) (GIBCO, USA). T24 cells (ATCC® HTB-4) were grown using McCoy 5A (modified) medium (Gibco, USA) supplemented with 10% BFS and 1x PSN. All cell lineages were kept in 5% CO_2_ atmosphere at 37°C.

Suspensions of each cell type were seeded into 24-well plates (1 × 10^5^ cells/ mL per well) and kept in culture according to the assay type. For qualitative assays, all cell lineages were cultivated for 2–5 days until they reached 80% confluency, except for Caco-2 cells, which were cultivated for 10 days to reach confluency, polarization, and differentiation. For quantitative assays, all cells were cultivated until they reached full confluency.

### Determination of the Bacterial Adherence Pattern

The adherence pattern was determined as described (Santos et al., 2019) using HeLa cells in 6 h of interaction in the presence of 2% D-mannose (Sigma - USA) to prevent Type 1 fimbria-mediated adhesion. Bacteria previously grown in LB for 18 h at 37°C were inoculated in 1 mL of cell media, at a 1:50 dilution, and incubated at 37°C for 3 h. The preparation was then washed with phosphate-buffered saline (PBS), fresh medium was added, and incubation proceeded for an additional 3 h. After the 6 h period, the cells were washed, fixed with methanol, stained with May Grünwald and Giemsa (Merck, New Jersey, USA) and evaluated by light microscopy (Hernandes et al., 2013). As controls, the *E. coli* prototype strains producing the LA (tEPEC E2348/69), AA (EAEC 042), and diffuse adhesion (DA) (DAEC C1845) patterns were used, as well as a non-adherent laboratory strain (*E. coli* HB101) (Hernandes et al., 2013).

### Fluorescence Actin Staining (FAS) Assay

The FAS test was used as an indirect approach to verify the ability of UPEC 252 to cause A/E lesions (Knutton et al., 1989). The test was performed in HeLa cells precisely as described for the determination of adhesion patterns. At the end of the assay, cells were fixed in 3% formaldehyde in PBS, permeabilized with 1% Triton X-100, and incubated with phalloidin for 20 min. Subsequently, coverslips were washed in PBS, mounted on glycerol, and observed under fluorescence microscopy (OLYMPUS BX60) (Olympus, Tokyo, Japan) with immersion lenses. As positive and negative controls, the *E. coli* E2348/69 (Jerse et al., 1990) and C1845 strains, respectively, were used.

The percentage of cells with pedestal produced by the UPEC 252 and EPEC E2348/69 strains was quantified in three different fields of view in a triplicated FAS assay. The results were shown by the means of the number of infected cells with F-actin accumulation foci (pedestals) ± SD. The unpaired bi-directional Student's *t*-test was used to compare means of the two strains, in which *P* ≤ 0.05 was considered statistically significant.

### Transmission Electron Microscopy

For Transmission Electron Microscopy (TEM), HeLa cells were grown onto ACLAR® film inserted into 24-wells plates, and subsequently subjected to an adhesion assay for 3 h as described above. In the end, the preparation was washed twice with 2% formaldehyde and 2.5% glutaraldehyde in 0.1 M sodium cacodylate buffer pH 7.2 and kept overnight in the same buffer. The material used was gradually infiltrated and embedded in gelatin capsules and allowed to polymerize for 48 h in an oven at 60°C; semi-thin sections (300–500 μm) were obtained and hot-stained with 1% toluidine blue. Finally, ultrathin sections (70 nm) were contrasted with uranium and lead citrate and visualized on a Jeol JEM 1200EX II transmission electron microscope. The typical EPEC E2348/69 and DAEC C1845 prototype strains were used as a positive and negative controls, respectively.

### Bacterial Interaction With Intestinal and Urinary Tract Cell Lineages

The ability of UPEC 252 to interact with intestinal (Caco-2 and LS174T) and urinary tract (T24 and HEK293) cell lineages was evaluated.

Adherence assays were performed as described above but in 3 h and the absence of D-mannose. For the quantitative adhesion assays, after the 3 h incubation period, cells were incubated for ~30 min with 1 mL sterile distilled water for cell lysis (Santos et al., 2019). Then, the suspension containing lysed cells and bacteria was subjected to serial dilutions and seeding onto MacConkey Agar to determine the number of colony-forming units (CFU) associated with the cells. The UPEC CFT073, aEPEC 4581-2 and non-adherent *E. coli* HB101 were included as controls. The One-way ANOVA followed by *post hoc* Tukey HSD test was used to compare the results obtained in this experiment.

The quantitative invasion assay was performed in duplicates, however, at the end of 3 h of incubation, one of the duplicates was washed with PBS, lysed by incubation with sterile distilled water for about 30 min, serially diluted, and plated onto MacConkey Agar to obtain the total number of bacteria interacting with the cells. The second duplicate was washed with PBS, incubated for an additional hour with DMEM containing 100 μg/mL gentamicin to kill the extracellular bacteria; after the incubation period, cells were washed, lysed, and the resulting preparations, diluted and seeded onto MacConkey agar to obtain the number of internalized bacteria (Luck et al., 2005). The invasion index was defined by the ratio of the total number of internalized CFUs by the total number of CFUs associated with the cells (externally and internally), expressed as a percentage. The invasive *Shigella flexneri* strain M90T was used as positive control while the non-invasive strain *Escherichia albertii* 1551-2::*eae*, mutated in the *eae* gene (Hernandes et al., 2008), was employed as a non-invasive control in all cellular lineages tested, except for the HEK293T cell lineage, where the DAEC C1845 strain was used as a negative control. All quantitative assays were performed in biological and experimental triplicates. The One-way ANOVA followed by *post hoc* Tukey HSD test was used to compare the results obtained in this experiment.

### Bacterial Resistance to the Human Serum Complement

To determine the resistance of UPEC 252 to the human serum complement, lyophilized human complement (Sigma, USA) reconstituted in PBS was used. Bacterial cells grown overnight in LB were washed twice and resuspended in PBS. The bacterial suspension was seeded onto MacConkey agar for CFU determination. The bacterial culture concentration was then adjusted to ~1 × 10^7^ CFU / mL in PBS. Then, 200 μl of the adjusted inoculum and 200 μl of human serum pool were mixed and incubated at 37°C for 4 h. Aliquots of the preparation were collected at three different times (30, 60, and 240 min), serially diluted, and plated onto MacConkey agar to obtain the survived bacterial counts. Simultaneously, another assay using serum inactivated by heating for 30 min at 56°C was performed. The experiments were carried out in biological triplicates, and the *E. coli* strain C600 and the *Enterobacter* spp. strain EB046 were used as serum sensitive and resistant controls, respectively (Keller et al., 1998). Additionally, the UPEC CFT073 and aEPEC 4581-2 strains were used as controls.

### Biofilm Formation

Analysis of biofilm formation was carried out as described previously (Lima et al., 2019). UPEC 252 was grown in LB at 37°C for 18 h. Then, 20 μl of the culture were added to 24-well polystyrene plates containing 1 mL of DMEM GlutaMax™ and incubated at 37°C for 24 or 48 h. After incubation, bacteria were washed with PBS, fixed overnight with 1 mL of formaldehyde (3%), and stained with 1% violet crystal. After successive washes, the dye impregnated in the cells was solubilized with 95% ethanol (Merck), supernatants were transferred to 96-well plates, and the optical density of the solution was measured in a spectrophotometer at 620 nm (Hernandes et al., 2013). The experiments were carried out in biological and experimental triplicates. The EAEC 042, UPEC CFT073, aEPEC 4581-2, and *E. coli* HB101 strains were used as controls. The One-way ANOVA followed by *post hoc* Tukey HSD test was used to compare the results obtained in this experiment.

### Antimicrobial Sensitivity Test

The diffusion disc technique was used as proposed by Bauer et al. (1966) to evaluate the antimicrobial susceptibility profile. The diameters of growth inhibition halos were measured and interpreted following the standards of the Brazilian Committee on Antimicrobial Susceptibility Testing - BrCAST breakpoints (EUCAST, 2020).

The antibiotics employed in this test were: amoxicillin-clavulanic acid (20 + 10 μg), aztreonam (30 μg), cefepime (30 μg), ceftazidime (10 μg), ceftriaxone (30 μg), meropenem (10 μg), imipenem (10 μg), ertapenem (10 μg), gentamicin (10 μg), amikacin (30 μg), tigecycline (15 μg), cefoxetin (30 μg), and ciprofloxacin (5 μg).

## Results

### Genetic Characterization of the LEE Region and Non-LEE Effectors in UPEC 252

UPEC 252 was originally reported to harbor the *eae* gene, which is a property characteristic of the EPEC and EHEC pathotypes (Abe et al., 2008). This fact raised the question of whether this strain would carry an intact LEE region, where the *eae* gene is located, and, therefore, could be potentially more virulent. Based on manual verification of the recently published genome sequence of UPEC 252 (Valiatti et al., 2019), we observed that the strain carried a complete LEE region (Table 1). Moreover, of all the 55 non-LEE effectors screened, only the *cif, nleB, nleB1, nleB2, nleC, nleE, nleF, nleG, nleH1, nleH2, espL*, and *espJ* genes were found.

**Table 1:** Genes that comprise the LEE region of UPEC252^a^.

**Gene**	**Coverage**	**%Coverage**	**%Identity**	**Accession**
*espG*	1–1,197/1,197	100.00	100.00	gi:260869665
*escE*	1–219/219	100.00	99.54	NP_290287
*escK*	1–199/199	100.00	99.50	AF022236.1
*escR*	1–654/654	100.00	100.00	gi:215488994
*escT*	1–777/777	100.00	99.87	gi:260869699
*etgA*	1–459/459	100.00	99.35	NP_290279
*grlA*	1–414/414	100.00	99.76	gi:260869695
*escC*	1–1,539/1,539	100.00	99.94	gi:215488986
*escJ*	1–573/573	100.00	99.83	gi:260869691
*espZ*	1–297/297	100.00	99.66	gi:260869689
*escV*	1–2,028/2,028	100.00	100.00	gi:260869687
*escO*	1–378/378	100.00	98.41	NP_290267
*sepQ/escQ*	1–918/918	100.00	93.14	NP_290265
*cesF*	1–363/363	100.00	99.45	gi:260869681
*tir*	1–1,656/1,656	100.00	99.94	gi:260869679
*eae*	1-2808/2808	100.00	100.00	gi:260869677
*sepL*	1–1,056/1,056	100.00	100.00	gi:260869675
*espD*	1–1,143/1,143	100.00	100.00	gi:260869673
*cesD2*	135–408/408	67.16	99.27	gi:260869671
*escG*	1–279/279	100.00	96.77	NP_290251
*espF*	1–504/789	63.88	90.71	gi:260869668
*escF*	1–222/222	100.00	100.00	gi:260869670
*espB*	1–963/963	100.00	100.00	gi:260869672
*espA*	1–579/579	100.00	100.00	gi:260869674
*escD*	1–1,221/1,221	100.00	99.84	gi:260869676
*cesT*	1–471/471	100.00	100.00	gi:260869678
*map*	1–612/612	100.00	99.84	gi:260869680
*espH*	1–510/510	100.00	100.00	gi:260869682
*escP*	1–276/276	100.00	98.19	NP_290266
*escN*	1–1,341/1,341	100.00	99.92	gi:260869686
*cesL*	1–354/354	100.00	99.44	NP_290270
*escI*	1–429/429	100.00	93.24	NP_290272
*sepD*	1–456/456	100.00	100.00	gi:260869692
*cesD*	1–456/456	100.00	100.00	gi:260869694
*grlR*	1–372/372	100.00	100.00	gi:260869696
*escU*	1–1,038/1,038	100.00	100.00	gi:260869698
*escS*	1–273/273	100.00	99.63	gi:291285070
*escL*	1–615/615	100.00	99.67	NP_290284
*cesAB*	1–324/324	100.00	99.69	gi:15804247
*ler*	1–390/390	100.00	99.74	gi:215488999
*rorf1*	1–272/272	100.00	90.81	AF022236.1

a *Summary of LEE genes identified by the ABRicate program. The percentage of coverage and identity displayed were the highest identified, comparing UPEC 252 with O157: H7 Sakai (EHEC), O127: H6 E2348/69 (EPEC), and O111: H- 11128 (EHEC). Most proteins presented belong to the E. coli O111:H- str 11128*.

By examining the sequence of the LEE region using the BLAST program, UPEC 252 showed identities ranging from 93 to 99% with EPEC, EHEC, and *E. albertii* strains isolated from different sources and countries (Table 2). The highest identity was found between UPEC 252 and aEPEC strain 13E0767 isolated from cattle in Germany, and eight EHEC strains, five of them belonging to the O111 serogroup, including the EHEC O111:H- strain 11128 (Table 2).

**Table 2:** Comparison of the LEE region of UPEC 252 with strains available at the NCBI.

**Strain**	**Isolation year**	**Place**	**Pathotype**	**Serogroup**	**Source**	**Identity**	**Access number**
*Escherichia coli* 13E0767	1998	Germany	EPEC	O156	Cattle	99.97%	CP020107.1
*Escherichia coli* FORC_042	2013	South Korea	EPEC	NA^a^	Pork meat	99.93%	CP025318.1
*Escherichia coli* 2013C-3304	2013	USA	EHEC	O71	Stool human	99.92%	CP027593.1
*Escherichia coli* O111 RM9322	2009	USA	EHEC	O111	Stream	99.92%	CP028117.1
*Escherichia coli* 2015C-3101	2014	USA	EHEC	O111	Stool human	99.92%	CP027221.1
*Escherichia coli* O111:H- 11128	2001	Japan	EHEC	O111	Stool human	99.92%	AP010960.1
*Escherichia coli* RM9975	2009	USA	EHEC	O111	Stool human	99.91%	CP028432.1
*Escherichia coli* 95JB1	1995	Australia	EHEC	O111	Stool human	99.91%	CP021335.1
*Escherichia coli* O157:H7 Sakai	1996	Japan	EHEC	O157	Stool human	97.46%	BA000007.3
*Escherichia coli* O127:H6 E2348/69	1969	United kingdom	EPEC	O127	Stool human	97.19%	FM180568.1
*Escherichia coli* O121 RM8352	2009	USA	EHEC	O121	Sediment^b^	93.17%	CP028110.1
*Escherichia coli* O121:H19 16-9255	2016	Canada	EHEC	O121	Flour	93.17%	CP022407.1
*Escherichia albertii* _EC06_170	2006	Japan	NA	NA	Human	93.20%	AP014857.1

a *NA – Information not available or not applicable*.

b *the strain was isolated from sediments from a stream*.

### Assessment of the LEE Region Functionality

**Figure 1:**
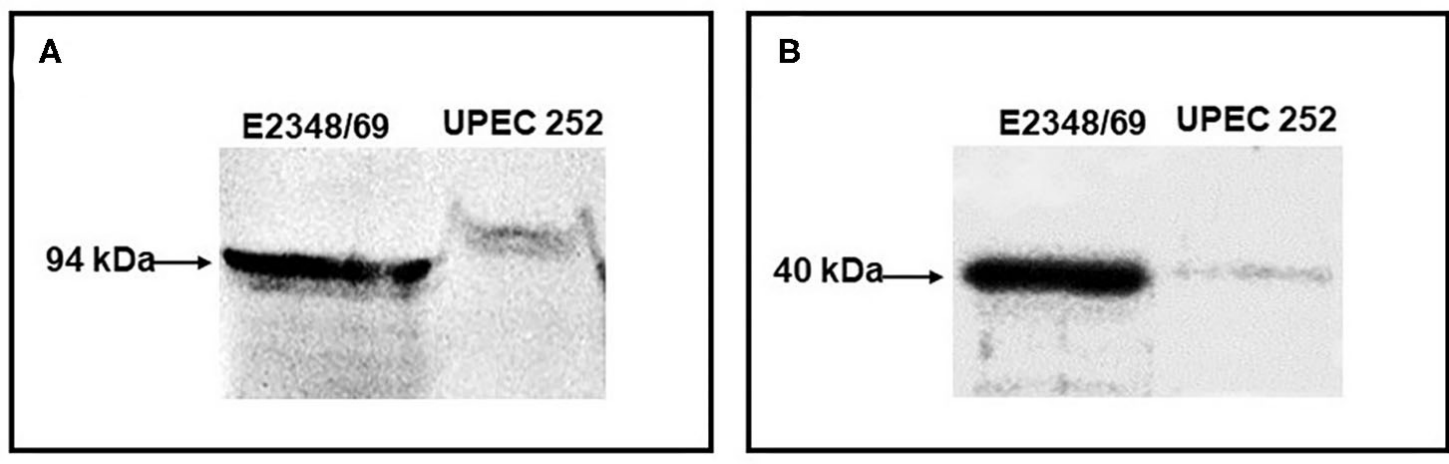
Immunodetection of Intimin and EspB. The production of the adhesin intimin and the secreted protein EspB were detected by immunoblotting. **(A)** Immunoblotting assay of UPEC 252 using an anti-intimin antibody (1:100) against the conserved region of the protein (Int388-667); **(B)** Immunoblotting assay of UPEC 252 using an anti-EspB antibody (1:3000). tEPEC strain E2348/69, positive control of production of intimin (94 kDa) and EspB (~ 40 kDa).

The functionality of the LEE was verified phenotypically. Initially, Intimin and EspB expression was confirmed by immunoblotting, in which both proteins exhibited their predicted molecular sizes (Figure 1).

**Figure 2:**
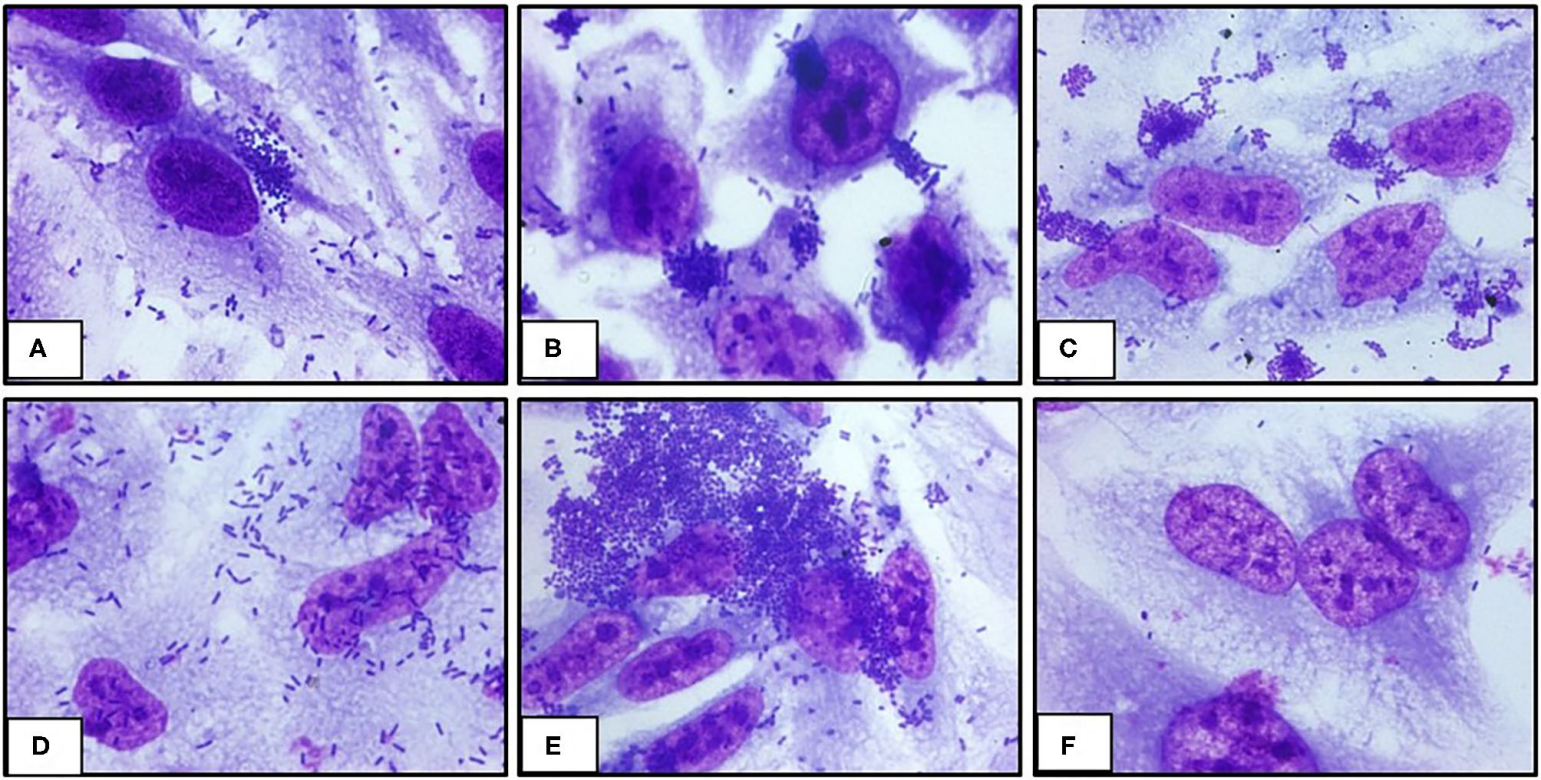
Adherence pattern of UPEC 252 to HeLa cells. The adherence pattern of the UPEC 252 **(A)** was obtained in a 6-h interaction assay in the presence of 2% D-mannose. Light microscopy images (microscopic magnification 1,000 ×) shows the production of loose bacterial microcolonies similar to the LAL (localized adherence-like) pattern of atypical EPEC. Controls: Localized adhesion: typical EPEC E2348/69 **(B)**; LAL: aEPEC 3991-1 **(C)**; Diffuse adhesion: DAEC C1845 **(D)**, Aggregative adhesion: EAEC 042 **(E)**; Non-adherent: *E. coli* HB101 **(F)**.

**Figure 3:**
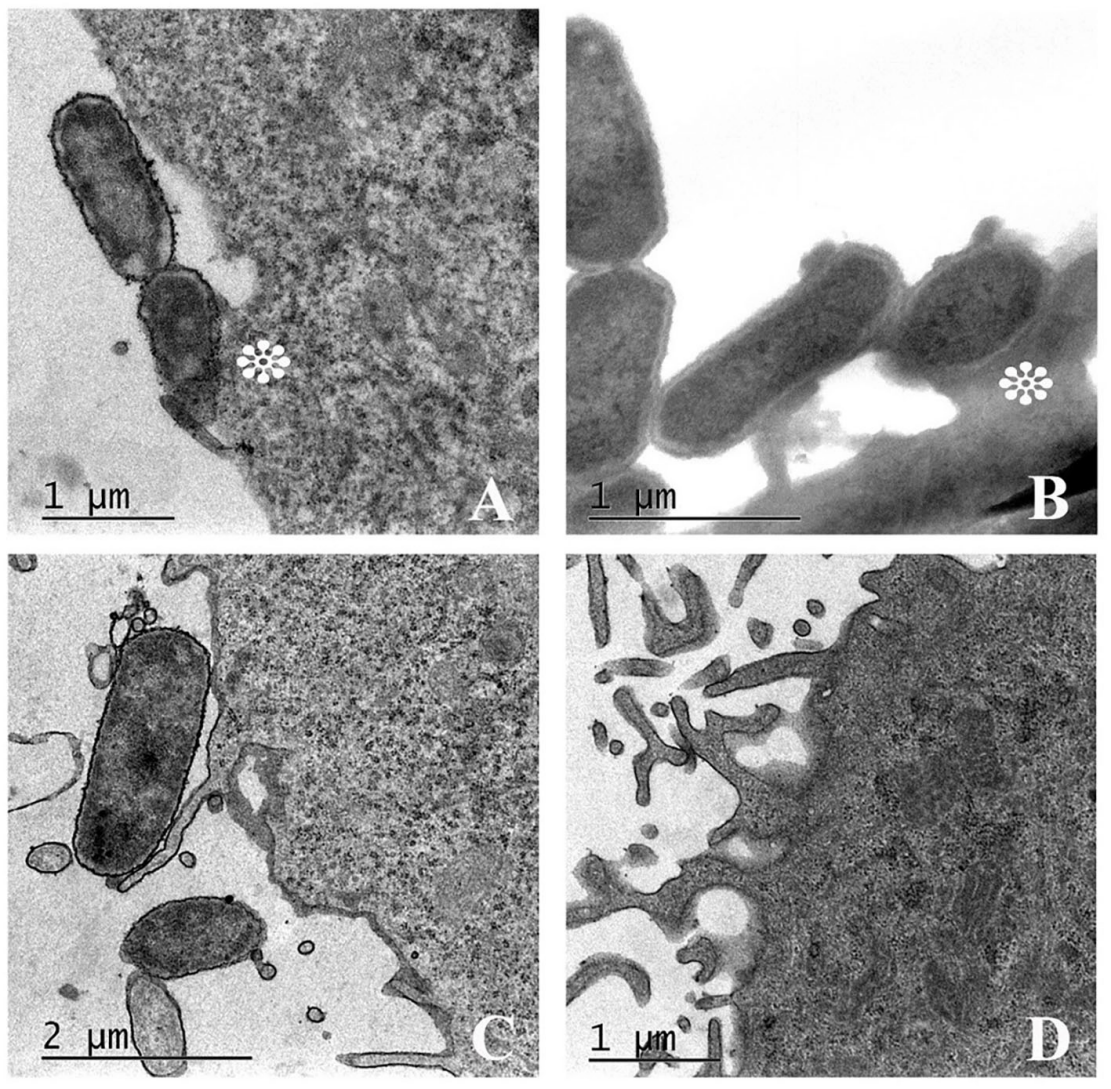
Attaching-effacing lesion promoted by UPEC 252. Transmission electron microscopy (TEM) of UPEC 252 evidencing cytoskeleton rearrangement and pedestal formation under the intimate bacterial adherence to HeLa cell. **(A)** A/E lesion produced by UPEC 252; **(B)** A/E lesion produced by positive control typical EPEC E2348/69; **(C)** Absence of A/E lesion in cells infected with the DAEC strain C1845, used as a negative control; **(D)** Non-infected cells. Asterisks indicate the pedestal-like structure formed under the adhered bacterium.

The ability of UPEC 252 to promote A/E lesion was then assessed in HeLa cells, where it presented a mixed adherence pattern either in the presence or absence of D-mannose, with occasional loose clusters resembling the Localized Adherence-Like (LAL) pattern (Figure 2), as well as bacteria diffusely attached to the cell surface, after 6 h of interaction. The ability of UPEC 252 to produce A/E lesion was then assessed by the FAS test, which revealed the capacity of the strain to mobilize actin. However, as observed by optical microscopy, actin mobilization occurred only occasionally, ~30% of the infected cells, 3-fold less of the mobilization caused by E2348/69 (Supplementary Figures 1, 2). Pedestal formation due to actin mobilization was found on the cells surface by TEM (Figure 3), showing that UPEC 252 bears a functional LEE capable of producing A/E lesion *in vitro*.

### UPEC 252 Genetic Background and Other Virulence-Associated Genes

**Figure 4:**
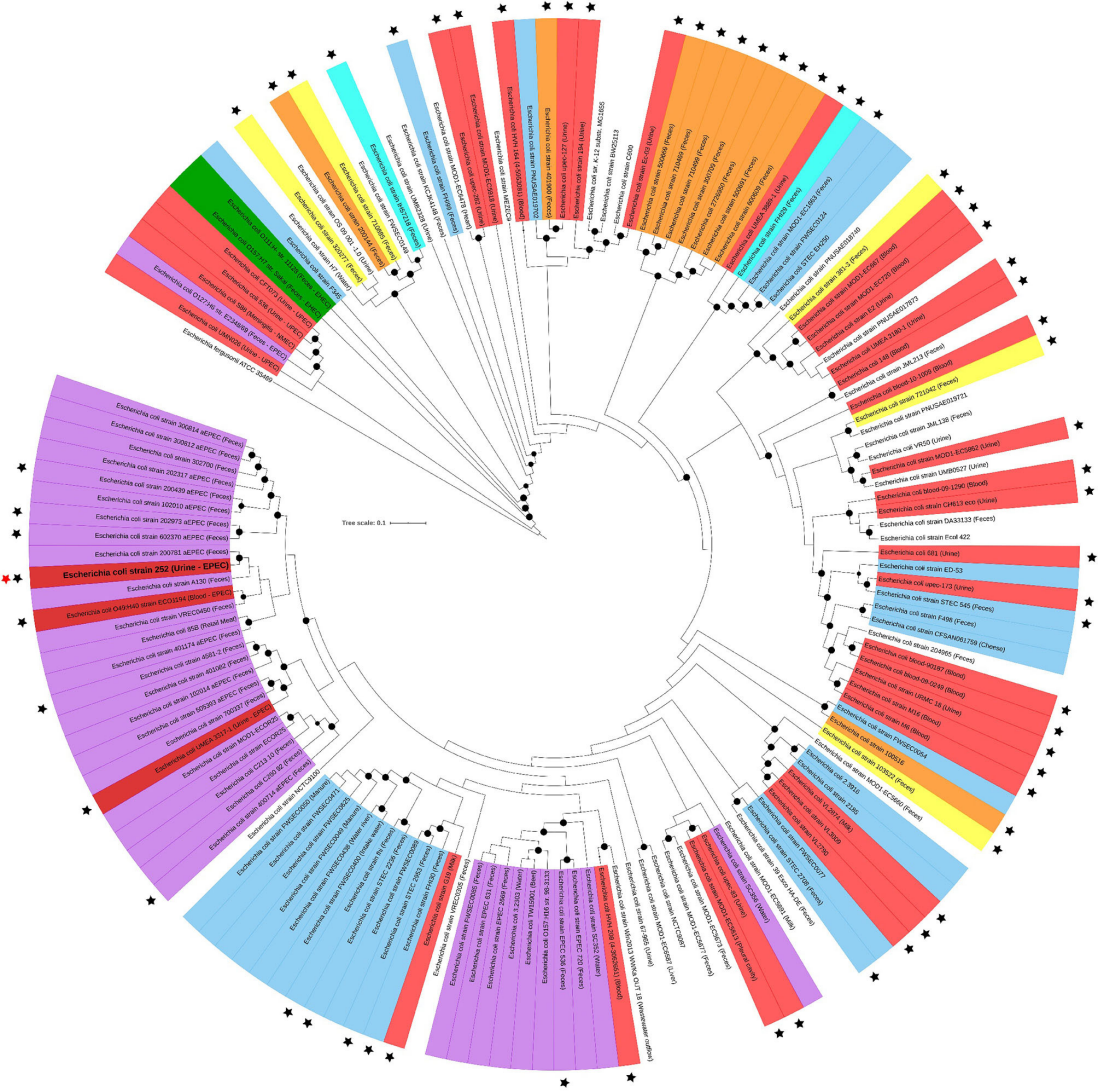
UPEC 252 phylogenetic tree. The phylogenetic tree was built with 140 public *E. coli* genomes belonging to ST10 using the codon-tree methodology of 1,000 single-copy proteins in the Maximum Likelihood-based matrix (RAxML). Labels are colored according to the *E. coli* pathotypes, which were determined by the presence of specific virulence factors or type of infection: red, extraintestinal pathogenic *E. coli* (strains isolated from extraintestinal infections); orange, enterotoxigenic *E. coli* (*elt* or *est* positive); light blue, Shiga toxin-producing *E. coli* (*stx* positive and *eae* and *escV* negative); green, enterohemorrhagic *E. coli* (*stx, eae* and *escV* positive) cyan, strains harboring *elt* and *stx* (ETEC/STEC); yellow, strains isolated from diarrhea but of unknown DEC pathotype; lilac, enteropathogenic *E. coli* (*eae* and *escV* positive, *stx* negative); dark red, extraintestinal pathogenic *E. coli* strains that, like UPEC 252, were isolated from extraintestinal infections but harbored EPEC virulence genes; uncolored, strains with no pathotype assignment or not confirmed as causing disease. Strains from known origins are indicated in parenthesis. Black stars indicate strains isolated from confirmed infections. UPEC 252 is labeled with a bold letter and indicated by a red star aside from the label. Bootstrap values higher than 90 are indicated by a black circle in the branch.

To understand the general genomic relationship of UPEC 252 with other *E. coli* isolates, phylogenetic analyses were performed, showing that it belongs to serotype O71:H40 and to phylogroup A. Multilocus sequence analysis conducted according to the Warwick scheme identified the strain as belonging to ST10. The phylogenetic tree built with strains of the ST10 showed that this ST is composed of strains from various DEC pathotypes, including strains presenting virulence encoding-genes of different DEC pathotypes and ExPEC strains isolated from different sites of infection (Figure 4). UPEC 252 was allocated in a cluster composed of aEPEC strains and two *E. coli* strains isolated from extraintestinal infections (Figure 4). Among the aEPEC strains with higher similarity, most were isolated from humans without intestinal infection (Figure 4 and Supplementary Table 1).

Regarding the virulence-encoding genes investigated, *in silico* analysis revealed the presence of 17 virulence genes/operons, as shown in Table 3, which are related to several pathogenicity mechanisms. Genes related to iron acquisition, serum resistance, and some adhesins were identified; although none of them are recognized as classically related to extraintestinal infections, except for *malX* (Table 3), they might contribute to the occurrence of extraintestinal infections. Several genes of different efflux pumps related to resistance to antimicrobials and other organic compounds were found in the UPEC 252 genome (Supplementary Table 2). However, no gene directly related to antimicrobial resistance was identified. Additionally, a phenotypic assay demonstrated that our strain was sensitive to all tested antimicrobials (Supplementary Table 3). The pangenome analyzes performed reinforced the phylogenetic relationship of UPEC 252 and aEPEC (Supplementary Figure 3), but did not evidence any gene that could be related to the establishment of UTI. Moreover, the genome alignment performed using progressiveMAUVE highlighted some insertion regions (composed of genes with unknown function or bacteriophages) in the UPEC 252 genome (Supplementary Figure 4) in comparison with EPEC/EHEC strains.

**Table 3:** Virulence-encoding genes detected in UPEC 252.

**Traits**	**Virulence factor**	**Gene/operon**
Adhesins/Invasins	*E. coli* laminin-binding fimbriae (ELF)	*elfACDG*
	Hemorrhagic *E. coli* pilus (HCP)	*hcpABC*
	Type I fimbriae	*fimABCDEFGHI*
	*E. coli* common pilus (ECP)	*ecpRABCDE*
	Curli adhesin	*csgABC-csgEFG*
	Intimin-like adhesin FdeC	*eaeH*
	IbeB (CusC) invasive protein	*cusC*
	EhaB autotransporter Protein	*ehaB*
Protectins	Surface exclusion protein	*traT*
	Increased serum survival	*iss*
	Colicin V production protein	*cvpA*
Iron acquisition system	Ferric hydroxamate system	*fhuACDB*
	Fe^3+^ uptake coprogen receptor	*fhuE*
	Ferric enterobactin receptor	*fepA*
Toxins	Hemolysin/cytolysin A	*hlyE*
	Cytoskeleton-binding toxin CbtA	*cbtA*
Pathogenicity island marker	PTS system maltose-specific	*malX*

*Virulence genes detected by VFBD and manually confirmed by BLAST*.

### UPEC 252 Is Resistant to Serum Complement Activity

**Figure 5:**
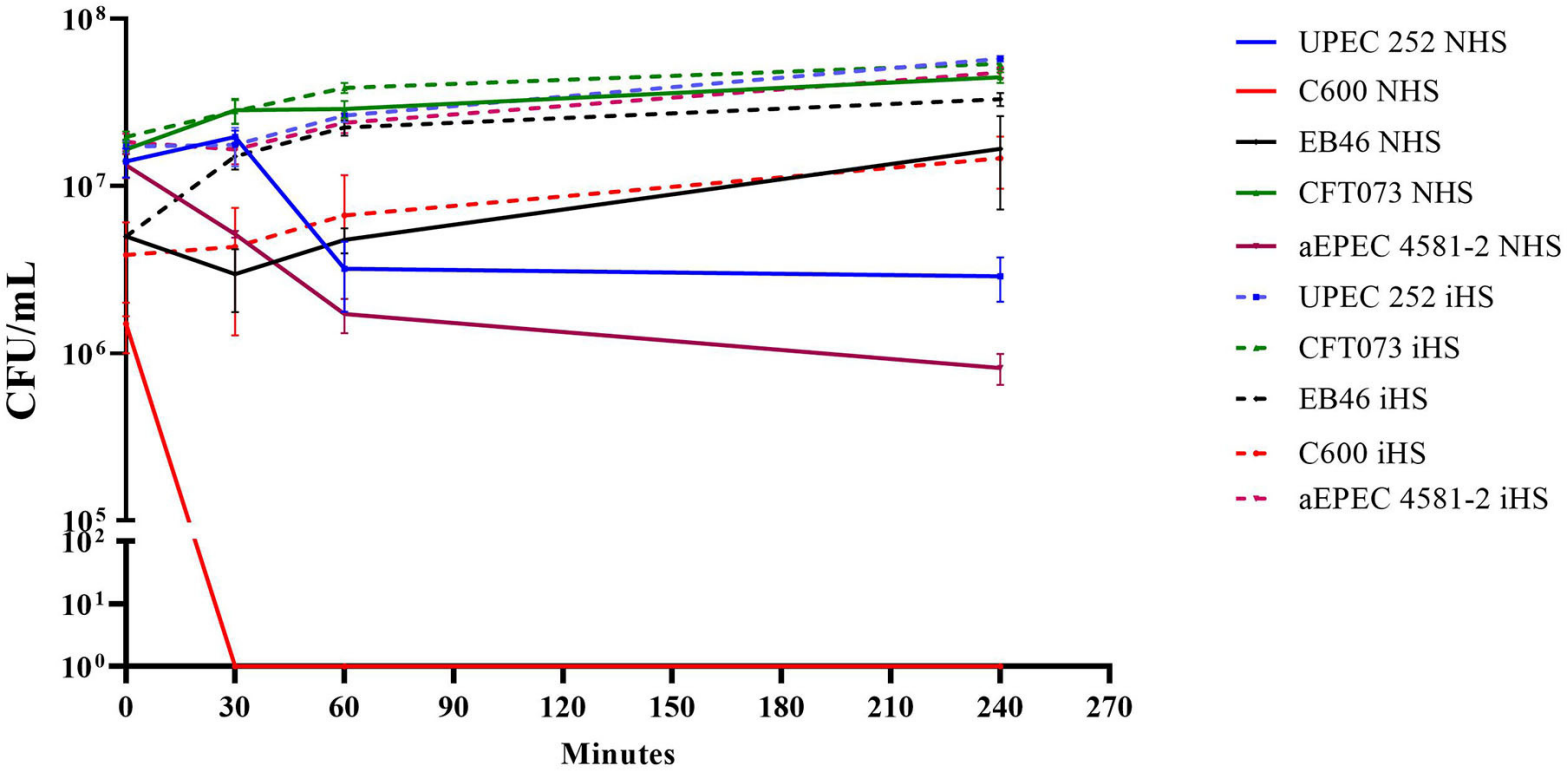
UPEC 252 serum resistance assay. Resistance to the bactericidal effect of the human serum was evaluated using a pool of human serum in PBS (50% of final serum concentration) in a 4-h assay. NHS, normal human serum; iHS, heat-inactivated human serum. The prototypical K-12 derived laboratory strain, *E. coli* C600, was used as a negative control, and the *Enterobacter* sp. strain EB046 was used as a positive control. Strains CFT073 and aEPEC4581 were also included in the experiment. The experiments were performed in biological triplicates.

As the analyses of the genome revealed the presence of the *traT* and *iss* genes, UPEC 252 was tested regarding its ability to resist the bactericidal power of human serum. Data demonstrated that UPEC 252 was serum resistant in the assayed condition, in which bacteria were incubated with serum complement in the physiologic concentration (50%) for at least 4 h, like the serum-resistant control *Enterobacter* sp. strain EB046 (Figure 5).

### UPEC 252 Does Not Produce Biofilm on Abiotic Surfaces

The ability to form biofilm on an abiotic surface (polystyrene) was tested at two different periods (24 and 48 h), however, under the conditions tested (cultivation in DMEM at 37°C) and regardless of the incubation period, the UPEC 252 was not able to form biofilm *in vitro* (Supplementary Figure 5).

### Evaluation of the Ability of UPEC 252 to Colonize and Invade Urinary and Intestinal Tract Cells *in vitro*

**Figure 6:**
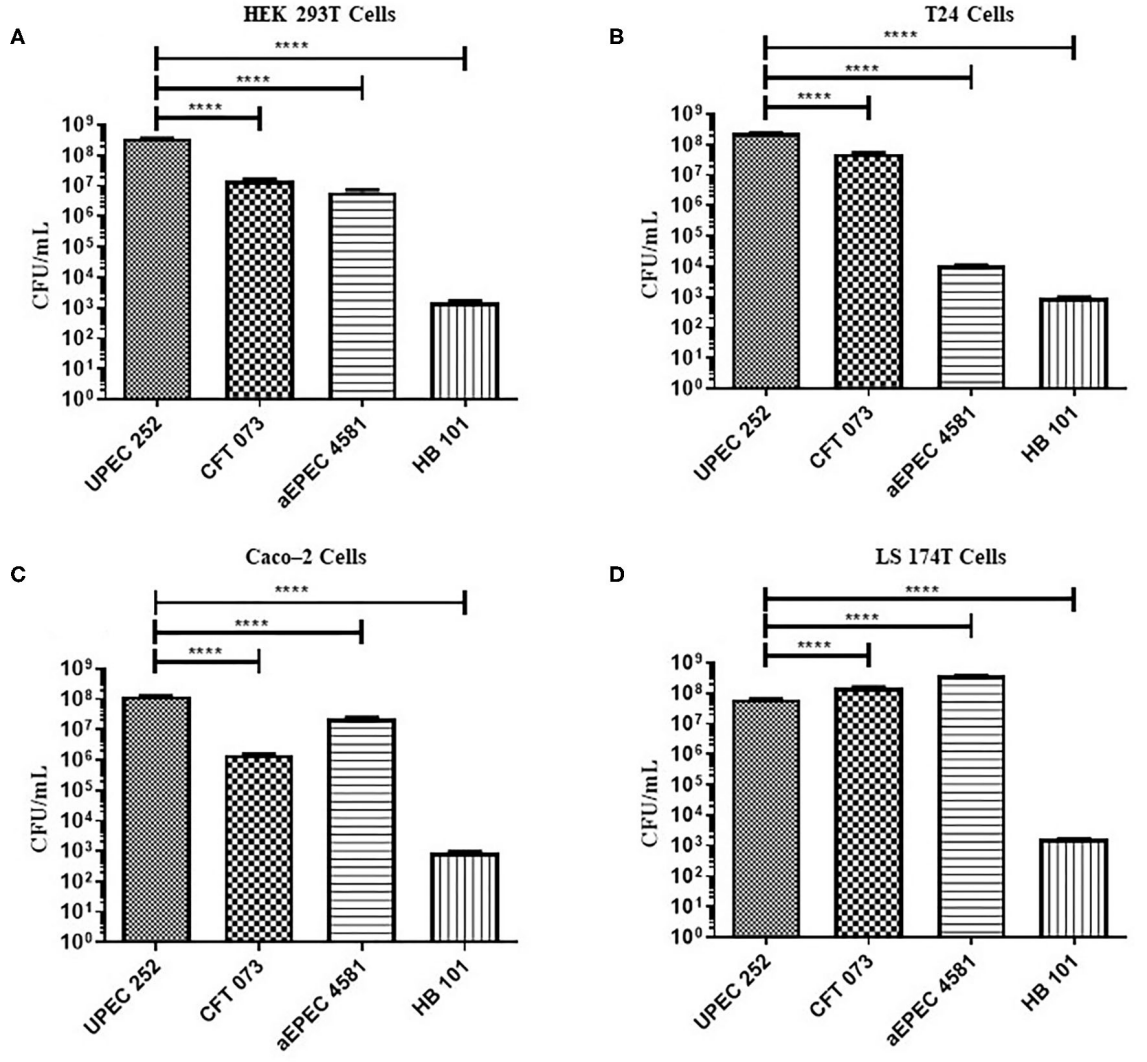
Quantitative adherence of UPEC 252 in different eukaryotic cell lineages. Quantitative assays were performed in a 3-h incubation period, in urinary (T24 and HEK 293T), and intestinal (Caco-2 and LS174T) tract cell lineages, in the absence of D-mannose. **(A)** HEK-293T cells; **(B)** T24 cells; **(C)** Caco-2 cells; **(D)** LS174T cells. The bacterial strains CFT073, aEPEC 4581-2, and *E. coli* HB101 were used as UPEC, aEPEC and poorly adherent controls, respectively. ****P ≤ 0.0001.

The ability of UPEC 252 to adhere to urinary (T24 and HEK 293T) and intestinal (Caco-2 and LS174T) epithelial cells was assessed using qualitative (Supplementary Figures 6, 7) and quantitative (Figure 6) adherence assays, with incubation periods of 3 h. UPEC 252 was able to adhere efficiently to all cell lines tested, and, when compared to UPEC (CFT073) and aEPEC (4581-2) controls, it showed higher adherence capacity in HEK293T, T24, and Caco-2 cells (*p* < 0.0001) than in the LS174T cell lineage (*p* < 0.001).

Comparing UPEC 252 adherence capacity among the diverse cell lineages used, the number of bacteria that adhered to the cells of the urinary tract was higher when compared to the cells of the intestinal tract (^***^ *p* < 0.01) (Supplementary Figure 8).

Invasion assays were performed to verify the ability of UPEC 252 to invade epithelial cells. To this end, HeLa, HEK293T, and Caco-2 cells were infected for 3 h in the absence of mannose. Despite recovering internalized bacterial counts in the order of 10^4^ CFU/mL, the invasion indexes observed were 0.028% (HeLa cells), 0.024% (HEK293T cells), and 0.05% (Caco-2 cells) (Supplementary Figures 9–11). These indexes were considerably low in comparison with the index of the classical invasive strain, *Shigella flexneri* M90T, used as a positive control.

## Discussion

In this study, we sought to perform a genotypic and phenotypic characterization of UPEC 252, which carried the *eae* gene (Falsetti, 1998; Abe et al., 2008) that is characteristic of the EPEC and EHEC pathotypes, what suggested that this strain could be capable of producing intestinal and extraintestinal infections in the same patient.

The alignment of the sequence of the LEE of UPEC 252 with the LEE of other strains revealed high similarities (more than 99.9% of identity) with aEPEC and EHEC strains isolated in Europe, Asia, Oceania, and North America. Interestingly, the strain whose genome showed the highest similarity was an aEPEC strain isolated in Germany in the same year as UPEC 252 (1998). In addition, we investigated if UPEC 252 harbored non-LEE genes that encode effector proteins that are secreted by A/E lesion-producing pathogens by a T3SS (Deng et al., 2012). Twelve among 55 known non-LEE genes (*cif, nleB, nleB*1*, nleB*2*, nleC, nleE, nleF, nleG, nleH*1, *nleH*2*, espL*, and *espJ*) were identified. It should be noted that the non-LEE proteins are not directly related to A/E lesion formation, but their activities may contribute to increased bacterial virulence by other pathways (reviewed by Gomes et al., 2016). The non-LEE genes found in UPEC 252 are related to several pathogenic mechanisms, such as inhibition of cell detachment and modulation of cell death (*cif* and *nleF*); inhibition of phagocytosis (*nleG, espJ, nleH*1, and *nleH*2), modulation of pro-inflammatory signaling (*nleB, nleB*1*, nleB*2*, nleC, nleE, nleG, nleH*1, and *nleH*2), and cytoskeleton modulation (*espL*) (Dean and Kenny, 2009; Vieira et al., 2010; Vossenkämper et al., 2011; Wong et al., 2011; Salvador et al., 2014).

Immunoblotting assays revealed that UPEC 252 produced intimin and secreted the EspB protein, thus suggesting that the LEE region was functional in this strain. Given the similarity of UPEC 252 with aEPEC strains (presence of the LEE region, and absence of pEAF and the *stx* genes), its adhesion pattern was investigated on HeLa cells, using prolonged assays (6-h assay) in the presence of D-mannose. Although UPEC 252 presented a non-characteristic adhesion pattern, it adhered randomly, forming loose microcolonies, which is the same pattern observed in some aEPEC strains. Thus, the presence of the LEE region in the UPEC 252 genome is probably contributing to the formation of the observed loose clusters, but further studies should be performed to confirm this hypothesis.

To corroborate the hypothesis of the ability of the UPEC 252 to produce A/E lesion, we employed the FAS test, which is commonly used as an indirect measure to determine the capacity of EPEC, EHEC, *E. albertii*, and *Citrobacter rodentium* strains to remodel the host cell cytoskeleton (Knutton et al., 1991; Lima et al., 2019; Tanabe et al., 2019). Although we have verified that UPEC 252 can promote actin accumulation in HeLa cells at the site of bacterial adhesion at only some fields and 3-fold less than a typical EPEC strain, TEM images revealed the occurrence of pedestal-like structures on the cell surface. Additional experiments are necessary to confirm further the ability of this strain to promote A/E lesions *in vivo* since it has been shown that not all LEE-positive strains that produce these lesions are FAS positive *in vitro* (Bai et al., 2008).

Genomic analyses have improved our understanding of how *E. coli* strains have evolved, revealing their genetic backgrounds, and highlighting the occurrence of horizontal gene transfer. Our phylogenetic analysis evidenced that UPEC 252 belonged to phylogroup A, ST10, and serotype O71:H40. *E. coli* strains of the ST10 have been frequently associated with UTI in several countries, including Brazil (Gonçalves et al., 2016; Hertz et al., 2016; Campos et al., 2018). Other studies have also reported the isolation of *E. coli* strains of the ST10 from the environment and food, as well as from bloodstream infections and diarrhea, evidencing the versatility of colonization of this ST (Arais et al., 2018; Liu et al., 2018; Mohsin et al., 2018; Yamaji et al., 2018).

In addition, our analysis showed that ST10 is composed of *E. coli* strains from various DEC pathotypes (ETEC, STEC, EPEC), as well as commensals and strains isolated from a variety of extraintestinal infections. Moreover, strains harboring combined virulence-encoding-genes used as diagnostic markers of the different DEC pathotypes (*e.g.*, ETEC/STEC) were identified among the ST10 strains. Furthermore, two strains isolated in Europe (Sweden and England) from extraintestinal infections and harboring the LEE region were detected. One of them was isolated in 1995 from the urine of an inpatient that developed urosepsis, while the other was isolated in a surveillance program from a bloodstream infection in 2008 (Kallonen et al., 2017). Altogether, these data corroborate with the hypotheses that some *E. coli* STs could be considered a “melting pot” that favors the emergence of hybrid strains, i.e., strains presenting concomitantly virulence markers that define different *E. coli* pathotypes (Gati et al., 2019). The ST10 complex could be a candidate for this emergence because it encompasses all *E. coli* pathotypes, and a variety of hybrid strains have been identified in this ST (STEC/ETEC, EAEC/UPEC, and EPEC/ExPEC).

In this context, two hypotheses were raised: UPEC 252 could be an ExPEC strain that acquired the LEE island, or an aEPEC strain that, over time, received ExPEC genes that allowed it to establish UTI. Our phylogenetic analyses supported the latter hypothesis because UPEC 252 is inserted into a cluster composed of aEPEC strains, suggesting that it has an EPEC genomic background. The diversity of aEPEC strains and their ability to acquire virulence genes by horizontal transfer were demonstrated by Hazen et al. (2017) through genome analyses. These authors showed that EPEC/ETEC hybrid strains were genetically closer to EPEC, and later acquired ETEC virulence genes. It is worth to mention that most aEPEC strains in the UPEC 252 cluster were isolated from the gut of asymptomatic human carriers, besides the two ExPEC strains that also harbored the LEE and were isolated from extraintestinal infection of non-diarrheic inpatients. These data suggested that this specific cluster could harbor strains with an attenuated diarrheagenic phenotype, which is reinforced by the few FAS positive signals produced by UPEC 252 in HeLa cells mentioned above. However, more studies are necessary to confirm this hypothesis because ST10 bears other EPEC clusters in which several pathogenic EPEC strains that had been isolated from diarrhea were detected (Ferdous et al., 2016; Arais et al., 2018; Santos et al., 2019). Besides the EPEC related genes, other virulence factors were identified in the genome of UPEC 252, among which genes that are involved in bacterial adherence to host cells, like *E. coli* laminin-binding fimbriae (ELF), *E. coli* common pilus (ECP), and Type 1 fimbriae. In UPEC strains, Type I fimbriae were demonstrated to be essential for adherence to the bladder epithelium (Paneth cells) at the early stage of UTI establishment (Connell et al., 1996; Sokurenko et al., 1998). Analysis of the genome of UPEC 252 also revealed the presence of the *iss* and *traT* genes, which confer to *E. coli* strains resistance to the bactericidal power of human serum (Binns et al., 1979) that is the first line of host defense (Walport, 2001). The serum resistance ability of UPEC 252 was confirmed in experiments conducted *in vitro*. This mechanism is essential for the pathogenicity of strains that reach the bloodstream since it allows bacteria to move across the whole human body, evading such a system and, consequently, aggravating the patient's clinical condition.

We also identified the presence of *mal*X in the genome of UPEC 252, which encodes enzyme II of the phosphotransferase system, and is used as the pathogenicity island marker of the UPEC prototype strain CFT073, whose relationship with UTI persistence and recurrence was previously demonstrated (Ejrnæs, 2011; Ejrnæs et al., 2011). It is noteworthy that *mal*X is essential for the persistence of *E. coli* strains in the intestinal tract of healthy babies (Östblom et al., 2011).

Biofilm production allows bacteria to persist in the host and can be associated with the gravity of the infection (Høiby et al., 2011). UPEC strains have been shown to present varying levels of biofilm production (Watts et al., 2010; Novais et al., 2012; Ponnusamy et al., 2012; Agarwal et al., 2013; Tapiainen et al., 2014; Flament-Simon et al., 2019). Herein, we showed that UPEC 252 is not capable of producing biofilm on abiotic surfaces under the conditions tested.

The ability of bacterial strains to adhere to and invade host cells are important mechanisms of virulence, which directly implicates increased pathogenicity since these processes are associated with persistent infections. We showed that UPEC 252 could adhere to different cell lines of the urinary and intestinal tracts *in vitro*. A higher affinity with the urinary tract epithelial cells was observed, which was expected, considering that it was isolated from a urinary tract infection. However, whether UPEC 252 can interact with the intestinal epithelium and cause disease in the intestinal tract remains to be investigated. Furthermore, the ability to invade the host cells allows bacteria to overcome the actions of the immune system and certain antibiotics (Lewis et al., 2016). Especially in female patients, UTIs often recur even after antibiotic therapy (Dielubanza and Schaeffer, 2011; Barber et al., 2013). Studies estimate that ~1-3rd of women up to 24 years old will be affected by UTI, and about 25% of these women will have at least one recurrent UTI in the following 6 months (Foxman, 2014). According to Lewis et al. (2016), recurrent UTIs are probably related to the invasiveness, persistence, and multiplication of bacteria within the urinary epithelium. Although the internalization rates of UPEC 252 were considerably lower than those of a classical invasive strain (*S. flexneri*), our findings evidenced the ability of this strain to invade the three different cell lines tested (HeLa, HEK293T, and Caco-2), suggesting that it may cause recurrent UTI, or even persistent diarrhea, since it was also able to invade differentiated intestinal Caco-2 cells. As in the present study, Owrangi et al. (2018) verified the ability of UPEC strains to adhere to and invade Caco-2 cells, with 10^4^ CFU being recovered from the intracellular compartment.

The occurrence in UPEC 252 of genes characteristic of DEC, the isolation of an symptomatic urinary tract infection, and the presence of ExPEC traits simultaneously suggests that it may cause infections in both the intestinal and extraintestinal tracts, and can be classified as a hybrid aEPEC/UPEC strain (Santos et al., 2020a). Likewise, Lara et al. (2017) identified two UPEC strains carrying EAEC and UPEC genes simultaneously. In the same study, an *E. coli* strain isolated from bacteremia with the same hybrid characteristics as the UPEC strains was also found. By analyzing the presence of specific genes of the DEC pathotypes, Salmani et al. (2016) identified UPEC strains that carried the *st, lt, agg*R, *inv*E, and *daa*D genes; however, none of them was positive for the *eae* gene. In contrast, Kim et al. (2018) found six UPEC strains of serogroups O2, O15, O18, and O25 that carried *eae*; however, these authors did not demonstrate whether these strains had complete and functional LEE regions.

In addition to our previous study (Abe et al., 2008), four other studies have reported the occurrence of EPEC/ExPEC hybrid strains (Toval et al., 2014; Kessler et al., 2015; Riveros et al., 2017; Lindstedt et al., 2018). In two of them, the strains were isolated from urinary tract infections and carried EPEC virulence genes but were devoid of the most common ExPEC virulence factors, like P fimbriae (Toval et al., 2014; Riveros et al., 2017). Interestingly, these strains also lacked the virulence genes used to classify the intrinsic virulence of pathogenic ExPEC strains, which is determined by the presence of at least two among the following virulence genes: *iuc/iut, pap, sfa, afa*, and *kpsMTII* (Johnson et al., 2003). In this context, these strains were similar to UPEC 252, whose genome carried a few ExPEC virulence-encoding genes. The other two studies reported on the occurrence of *E. coli* strains from diarrheal patients that harbored the ExPEC intrinsic virulence encoding-genes; in one of these patients, bacteremia and multiorgan dysfunction development were reported (Kessler et al., 2015; Lindstedt et al., 2018). Notably, these reports included strains from three different STs (ST12, ST28, and ST2018) of phylogroups B2 (Toval et al., 2014; Kessler et al., 2015; Lindstedt et al., 2018) and one strain from phylogroup A, whose ST was not determined (Riveros et al., 2017).

Although some *E. coli* clones of ST10 are recognized as ExPEC strains involved in various human extraintestinal infections (Bert et al., 2010; Manges et al., 2017; Yamaji et al., 2018), they do not fulfill the molecular criteria to be recognized as intrinsic virulent (Johnson et al., 2003, 2015) or uropathogenic (Spurbeck et al., 2012) strains. The absence of the most common traits related to ExPEC virulence does not limit their capacity to cause more than 10% of infections in some countries (Manges and Johnson, 2015; Campos et al., 2018; Manges et al., 2019) nor their importance as a recognized foodborne cause of extraintestinal infections that has been continuously reported worldwide (Campos et al., 2018; Yamaji et al., 2018; Manges et al., 2019). Additionally, we identified two other strains that harbored the LEE and were isolated from extraintestinal infections in the same phylogenetic cluster of UPEC 252.

Despite the frequent reports about *E. coli* from ST10 harboring multidrug resistance genes, including *mcr-1, bla*_*NDM*_, and *bla*_*CTX*_(Zhang et al., 2017; García et al., 2018; Mohsin et al., 2018; dos Anjos et al., 2019), UPEC 252 was shown to be sensitive to the tested antimicrobials. The detection of some resistance-related genes in UPEC 252 suggests that there is a potential phenotype of resistance for this strain, which perhaps was not detected due to the lack of other essential genes and /or gene expression.

Finally, the data presented here contribute to the discussion about hybrid *E. coli* strains, more specifically, hybrid strains of UPEC, which have additional virulence mechanisms that are characteristic of other *E. coli* pathotypes. Therefore, hybrid UPEC strains may emerge as important pathogens in the world scenario, and further studies are necessary to better understand their virulence mechanisms, so that prevention, diagnosis, and treatment can be more appropriately addressed.

## Conclusion

UPEC 252 strain exhibits characteristics of ExPEC, EPEC and EHEC strains, along with the ability to adhere to and invade cells of the urinary and intestinal tracts *in vitro*. Our findings suggest that UPEC 252 is an atypical EPEC strain that emerges as a hybrid strain (aEPEC/UPEC), which could colonize new niches and potentially cause intestinal and extraintestinal infections.

## Data Availability Statement

The reads used for UPEC 252 genome assembly were deposited in the Sequence Read Archive (SRA) at NCBI under the accession number SRR9317828, and the whole-genome sequences (WGS) were deposited in the GenBank database under the accession number VFST00000000.

## Ethics Statement

This work was evaluated and approved by the local Research Ethics Committee of the Federal University of São Paulo-UNIFESP/São Paulo Hospital, under CEP N 8580081117. Written and informed consent was not required in accordance with local guidelines.

## Author Contributions

TG, TV, RMS, AS, and FS conceptualized the study. TV, AS, FS, EC, and TG contributed to the formal analysis. TG and TV were responsible for funding acquisition. TV, FS, AS, JN, and EC carried out the investigation. TV, FS, AS, JN, EC, and RS worked on the methodology and validated the study. TG helped with the project administration. TG and RMS supervised the study. TV, FS, and AS wrote the original draft. TV, FS, AS, JN, EC, and TG reviewed and edited the manuscript. All authors read and approved the final manuscript.

## Conflict of Interest

The authors declare that the research was conducted in the absence of any commercial or financial relationships that could be construed as a potential conflict of interest.

## Footnotes

^1^Seemann, T. *ABRicate*. Available online at: https://github.com/tseemann/abricate
