# Supplementary material for: Genetic and Virulence Characteristics of a Hybrid Atypical Enteropathogenic and Uropathogenic Escherichia coli (aEPEC/UPEC) Strain
Source: Front Cell Infect Microbiol. 2020 Sep 29;10:492. doi: 10.3389/fcimb.2020.00492 (PMC7550682; doi:10.3389/fcimb.2020.00492)
Supplement: Supplementary file 1 [file Data_Sheet_1.docx]

Supplementary Material


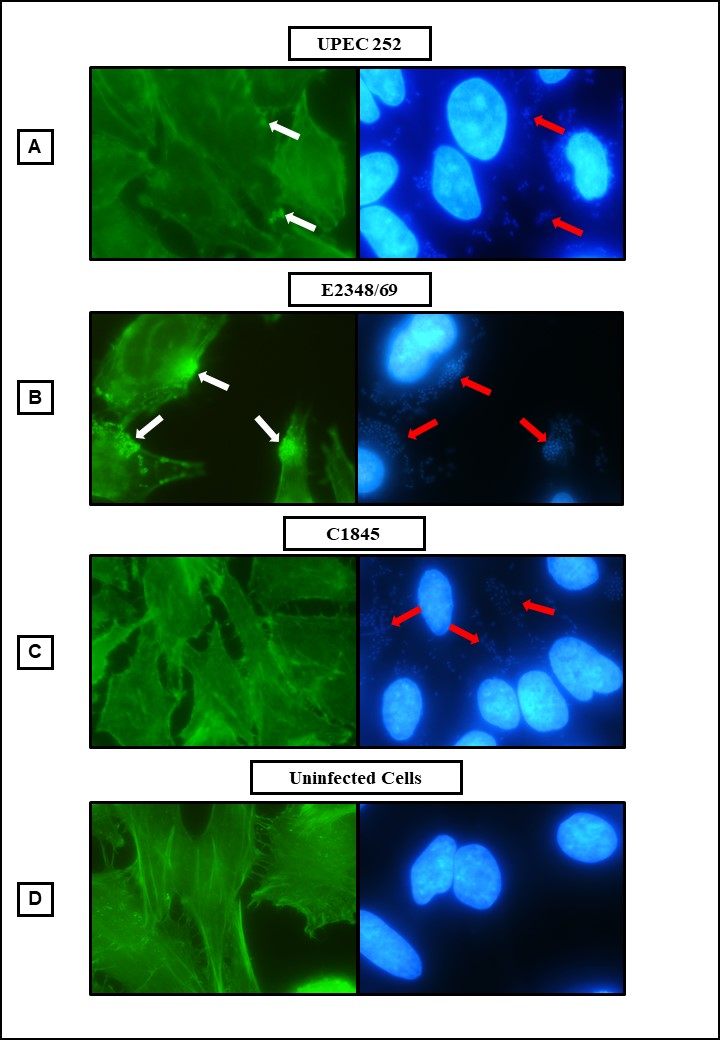


**Figure S1. Actin polymerization identification test (Fluorescence Actin Staining, FAS), in HeLa cells infected for 3 hours with UPEC 252.** Positive control: tEPEC E2348/69. Negative control: *E. coli* C1845. (Microscopic magnification of 1,000 x). Actin: phalloidin conjugated to fluorescein (Green). DNA: DAPI (Blue). The white arrows indicate the presence of actin accumulation, suggesting the formation of the A/E lesion. The red arrows indicate the site of bacterial adherence.

**Figure S2.**  **Cells with pedestals produced by the UPEC 252 and typical EPEC2348/69 strains during interaction with HeLa cells.** The percentage of the number of cells with pedestals was assessed in three different fields of three different preparations. The unpaired bi-directional Student’s t-test was used to compare means of the two strains, in which P- value ≤ 0.05 was considered statistically significant. ** P ≤ 0.01.

| **Table S1.** *Escherichia coli* strains used to perform phylogenetic analysis, their isolation sources and pathotypes | | | | | | | | | |
| --- | --- | --- | --- | --- | --- | --- | --- | --- | --- |
| **Strain (serotype)** | **Assembly or Biosample Accession Number** | **Phylogroup** | **MLST** | **Isolation source** | **Host** | **Isolated from infection** | **Pathotype ^a^** | **Sequencing reference** | **Observation** |
| E2348/69 (O127:H6) | [GCA_000026545.1](http://www.ncbi.nlm.nih.gov/assembly/GCA_000026545.1) | B2 | 15 | Stool | Human | Yes | EPEC | [18952797](https://www.ncbi.nlm.nih.gov/pubmed/18952797) |  |
| CFT073 | GCA_000007445.1 | B2 | 73 | Urine | Human | Yes | ExPEC | [12471157,2182540](http://www.ncbi.nlm.nih.gov/pubmed/12471157,2182540) |  |
| 536 | GCA_000013305.1 | B2 | 127 | Urine | Human | Yes | ExPEC | [16879640](http://www.ncbi.nlm.nih.gov/pubmed/16879640) |  |
| S88 | GCA_000026285.1 | B2 | 95 | Meningitis | Human | Yes | ExPEC | [19165319,19307211](http://www.ncbi.nlm.nih.gov/pubmed/19165319,19307211) |  |
| UMN026 | GCA_000026325.1 | D | 597 | Urine | Human | Yes | ExPEC | [19165319](http://www.ncbi.nlm.nih.gov/pubmed/19165319) |  |
| Sakai (O157:H7) | [GCA_000008865.1](http://www.ncbi.nlm.nih.gov/assembly/GCA_000008865.1) | E | 11 | Stool | Human | Yes | EHEC | [9628576, +4](https://www.ncbi.nlm.nih.gov/pubmed/9628576,10734605,11108008,11111050,11258796) |  |
| 11128 (O111:H-) | GCA_000010765.1 | B1 | 16 | Stool | Human | Yes | EHEC | [19815525](https://pubmed.ncbi.nlm.nih.gov/19815525/) |  |
| upec-127 | [GCA_000780965.1](http://www.ncbi.nlm.nih.gov/assembly/GCA_000780965.1) | A | 10 | Urine | Human | Yes | ExPEC | [25373147](https://www.ncbi.nlm.nih.gov/pubmed/25373147) |  |
| 401900 | [GCF_001912105.1](http://www.ncbi.nlm.nih.gov/assembly/GCF_001912105.1) | A | 10 | Stool | Human | Yes | -- |  |  |
| PNUSAE019702 | [GCA_003761285.1](http://www.ncbi.nlm.nih.gov/assembly/GCA_003761285.1) | A | 10 | M.D. | M.D. | M.D. | STEC | [218110](https://www.ncbi.nlm.nih.gov/bioproject/218110) |  |
| 194 | [SAMN05774074](http://www.ncbi.nlm.nih.gov/biosample/SAMN05774074) | A | 10 | Urine | Human | Yes | ExPEC |  |  |
| Ec-03 | [GCF_001893865.1](http://www.ncbi.nlm.nih.gov/assembly/GCF_001893865.1) | A | 10 | Urine | Human | Yes | ExPEC |  | Inpatient with chronic diarrhea |
| 500669 | [GCA_003981165.1](http://www.ncbi.nlm.nih.gov/assembly/GCA_003981165.1) | A | 10 | Stool | Human | Yes | ETEC |  |  |
| 710469 | [GCA_003981355.1](http://www.ncbi.nlm.nih.gov/assembly/GCA_003981355.1) | A | 10 | Stool | Human | Yes | ETEC |  |  |
| 710499 | [GCA_003980495.1](http://www.ncbi.nlm.nih.gov/assembly/GCA_003980495.1) | A | 10 | Stool | Human | Yes | ETEC |  |  |
| 300709 | [GCA_003956305.1](http://www.ncbi.nlm.nih.gov/assembly/GCA_003956305.1) | A | 10 | Stool | Human | Yes | ETEC |  |  |
| 600609 | [GCA_003981745.1](http://www.ncbi.nlm.nih.gov/assembly/GCA_003981745.1) | A | 10 | Stool | Human | Yes | ETEC |  |  |
| 2726950 | [GCA_000356145.1](http://www.ncbi.nlm.nih.gov/assembly/GCA_000356145.1) | A | 10 | Stool | Human | Yes | ETEC |  |  |
| 500691 | [GCA_003981835.1](http://www.ncbi.nlm.nih.gov/assembly/GCA_003981835.1) | A | 10 | Stool | Human | Yes | ETEC |  |  |
| MOD1-EC5918 | [GCA_002534875.1](http://www.ncbi.nlm.nih.gov/assembly/GCA_002534875.1) | A | 10 | Urine | Dog | Yes | ExPEC | [29242221](https://www.ncbi.nlm.nih.gov/pubmed/29242221) |  |
| MEZEC9 | [GCA_005938725.1](http://www.ncbi.nlm.nih.gov/assembly/GCA_005938725.1) | A | 10 | M.D. | Sheep | M.D. | -- |  |  |
| MOD1-EC6478 | [GCA_002485665.1](http://www.ncbi.nlm.nih.gov/assembly/GCA_002485665.1) | A | 10 | Heart | Chicken | M.D. | -- |  |  |
| upec-282 | [GCA_000777535.1](http://www.ncbi.nlm.nih.gov/assembly/GCA_000777535.1) | A | 10 | Urine | Human | Yes | ExPEC | [25373147](https://www.ncbi.nlm.nih.gov/pubmed/25373147) |  |
| HVH 164 [4-5953081] | [GCA_000458495.1](http://www.ncbi.nlm.nih.gov/assembly/GCA_000458495.1) | A | 10 | Blood | Human | Yes | ExPEC |  |  |
| FHI99 | [GCA_000951935.1](http://www.ncbi.nlm.nih.gov/assembly/GCA_000951935.1) | A | 10 | Stool | Human | Yes | STEC |  |  |
| KCJK4148 | [GCA_002324495.1](http://www.ncbi.nlm.nih.gov/assembly/GCA_002324495.1) | A | 10 | Stool | M.D. | M.D. | -- |  |  |
| F345 | [GCA_004767035.1](http://www.ncbi.nlm.nih.gov/assembly/GCA_004767035.1) | A | 10 | Stool | Pig | No | STEC | [15574914](https://www.ncbi.nlm.nih.gov/pubmed/15574914) |  |
| UMB2328 | [GCA_003886545.1](http://www.ncbi.nlm.nih.gov/assembly/GCA_003886545.1) | A | 10 | Urine | Human | M.D. | -- |  |  |
| OS_09_001_-1.0 | [GCA_002087785.1](http://www.ncbi.nlm.nih.gov/assembly/GCA_002087785.1) | A | 10 | Urine | Human | M.D. | -- |  |  |
| H7 | [GCF_001900455.1](http://www.ncbi.nlm.nih.gov/assembly/GCF_001900455.1) | A | 10 | Water river | NA | No | -- |  |  |
| 320277 | [GCA_003982555.1](http://www.ncbi.nlm.nih.gov/assembly/GCA_003982555.1) | A | 10 | Stool | Human | Yes | -- |  |  |
| IH57218 | [GCF_001264215.1](http://www.ncbi.nlm.nih.gov/assembly/GCF_001264215.1) | A | 10 | Stool | Human | Yes | STEC/ETEC |  |  |
| FWSEC0149 | [GCA_005044235.1](http://www.ncbi.nlm.nih.gov/assembly/GCA_005044235.1) | A | 10 | M.D. | Human | M.D. | -- |  |  |
| 200144 | [GCF_001911595.1](http://www.ncbi.nlm.nih.gov/assembly/GCF_001911595.1) | A | 10 | Stool | Human | Yes | ETEC |  |  |
| 710885 | [GCA_003981345.1](http://www.ncbi.nlm.nih.gov/assembly/GCA_003981345.1) | A | 10 | Stool | Human | Yes | -- |  |  |
| UMEA 3889-1 | [GCA_000461755.1](http://www.ncbi.nlm.nih.gov/assembly/GCA_000461755.1) | A | 10 | Urine | Human | Yes | ExPEC |  |  |
| FHI29 | [GCF_000942915.1](http://www.ncbi.nlm.nih.gov/assembly/GCF_000942915.1) | A | 10 | Stool | Human | Yes | STEC/ETEC |  |  |
| MOD1-EC1663 | [GCA_002459965.1](http://www.ncbi.nlm.nih.gov/assembly/GCA_002459965.1) | A | 10 | Stool | Human | Yes | STEC | [29242221](https://www.ncbi.nlm.nih.gov/pubmed/29242221) |  |
| STEC_EH250 | [GCA_000225145.2](http://www.ncbi.nlm.nih.gov/assembly/GCA_000225145.2) | A | 10 | M.D. | M.D. | M.D. | STEC |  |  |
| FWSEC0124 | [GCA_005044585.1](http://www.ncbi.nlm.nih.gov/assembly/GCA_005044585.1) | A | 10 | M.D. | M.D. | M.D. | STEC |  |  |
| PNUSAE018740 | [GCA_003782245.1](http://www.ncbi.nlm.nih.gov/assembly/GCA_003782245.1) | A | 10 | M.D. | M.D. | M.D. | -- |  |  |
| 381-3 | [GCF_000948005.1](http://www.ncbi.nlm.nih.gov/assembly/GCF_000948005.1) | A | 10 | Stool | Human | Yes | -- | [25656624](https://www.ncbi.nlm.nih.gov/pubmed/25656624) |  |
| MOD1-EC667 | [GCA_002466925.1](http://www.ncbi.nlm.nih.gov/assembly/GCA_002466925.1) | A | 10 | Blood | Human | Yes | ExPEC |  |  |
| MOD1-EC720 | [GCA_002456415.1](http://www.ncbi.nlm.nih.gov/assembly/GCA_002456415.1) | A | 10 | Blood | Human | Yes | ExPEC |  |  |
| E2 | [GCF_001693555.1](http://www.ncbi.nlm.nih.gov/assembly/GCF_001693555.1) | A | 10 | Urine | Human | Yes | ExPEC |  |  |
| PNUSAE017873 | [GCA_003770225.1](http://www.ncbi.nlm.nih.gov/assembly/GCA_003770225.1) | A | 10 | M.D. | M.D. | M.D. | -- |  |  |
| UMEA 3180-1 | [GCA_000460635.1](http://www.ncbi.nlm.nih.gov/assembly/GCA_000460635.1) | A | 10 | Urine | Human | Yes | ExPEC |  |  |
| PNUSAE019721 | [GCA_003776245.1](http://www.ncbi.nlm.nih.gov/assembly/GCA_003776245.1) | A | 10 | M.D. | M.D. | M.D. | -- |  |  |
| 721042 | [GCA_003980375.1](http://www.ncbi.nlm.nih.gov/assembly/GCA_003980375.1) | A | 10 | Stool | Human | Yes | -- |  |  |
| UMB0527 | [GCA_003892535.1](http://www.ncbi.nlm.nih.gov/assembly/GCA_003892535.1) | A | 10 | Urine | Human | M.D. | -- |  |  |
| MOD1-EC5862 | [GCA_002548165.1](http://www.ncbi.nlm.nih.gov/assembly/GCA_002548165.1) | A | 10 | Urine | Human | Yes | ExPEC |  |  |
| VR50 | [GCF_000968515.1](http://www.ncbi.nlm.nih.gov/assembly/GCF_000968515.1) | A | 10 | Urine | Human | No | -- |  |  |
| JML138 | [GCA_005387225.1](http://www.ncbi.nlm.nih.gov/assembly/GCA_005387225.1) | A | 10 | Stool | Human | No | -- |  |  |
| blood-10-1009 | [GCA_000779005.1](http://www.ncbi.nlm.nih.gov/assembly/GCA_000779005.1) | A | 10 | Blood | Human | Yes | ExPEC | [25373147](https://www.ncbi.nlm.nih.gov/pubmed/25373147) |  |
| JML213 | [GCA_005388505.1](http://www.ncbi.nlm.nih.gov/assembly/GCA_005388505.1) | A | 10 | Stool | Human | No | -- |  |  |
| 148 | [GCA_000511425.1](http://www.ncbi.nlm.nih.gov/assembly/GCA_000511425.1) | A | 10 | Blood | Human | Yes | ExPEC |  |  |
| blood-09-1290 | [GCA_000781915.1](http://www.ncbi.nlm.nih.gov/assembly/GCA_000781915.1) | A | 10 | Blood | Human | Yes | ExPEC | [25373147](https://www.ncbi.nlm.nih.gov/pubmed/25373147) |  |
| CH613_eco | [SAMN05425592](http://www.ncbi.nlm.nih.gov/biosample/SAMN05425592) | A | 10 | Urine | Human | Yes | ExPEC |  |  |
| DA33133 | [GCA_003180975.1](http://www.ncbi.nlm.nih.gov/assembly/GCA_003180975.1) | A | 10 | Stool | Human | M.D. | -- |  |  |
| Ecol_422 | [GCF_002012185.1](http://www.ncbi.nlm.nih.gov/assembly/GCF_002012185.1) | A | 10 | M.D. | Human | M.D. | -- |  |  |
| F498 | [GCA_002319165.1](http://www.ncbi.nlm.nih.gov/assembly/GCA_002319165.1) | A | 10 | Feces | Pig | No | STEC |  |  |
| CFSAN061759 | [GCA_003399875.1](http://www.ncbi.nlm.nih.gov/assembly/GCA_003399875.1) | A | 10 | Raw milk cheese | NA | No | -- |  |  |
| 681 | [GCA_000503335.1](http://www.ncbi.nlm.nih.gov/assembly/GCA_000503335.1) | A | 10 | Urine | Human | Yes | ExPEC |  |  |
| ED-53 | [GCA_002319255.1](http://www.ncbi.nlm.nih.gov/assembly/GCA_002319255.1) | A | 10 | M.D. | Pig | No | STEC |  |  |
| STEC 545 | [GCF_001609745.1](http://www.ncbi.nlm.nih.gov/assembly/GCF_001609745.1) | A | 10 | Stool | Human | Yes | STEC |  |  |
| upec-173 | [GCA_000779955.1](http://www.ncbi.nlm.nih.gov/assembly/GCA_000779955.1) | A | 10 | Urine | Human | Yes | ExPEC | [25373147](https://www.ncbi.nlm.nih.gov/pubmed/25373147) |  |
| 204965 | [GCF_001940485.1](http://www.ncbi.nlm.nih.gov/assembly/GCF_001940485.1) | A | 10 | Stool | Human | No | -- |  |  |
| blood-90187 | [GCA_000779155.1](http://www.ncbi.nlm.nih.gov/assembly/GCA_000779155.1) | A | 10 | Blood | Human | Yes | ExPEC | [25373147](https://www.ncbi.nlm.nih.gov/pubmed/25373147) |  |
| blood-09-0249 | [GCA_000782275.1](http://www.ncbi.nlm.nih.gov/assembly/GCA_000782275.1) | A | 10 | Blood | Human | Yes | ExPEC | [25373147](https://www.ncbi.nlm.nih.gov/pubmed/25373147) |  |
| URMC_18 | [GCA_004568035.1](http://www.ncbi.nlm.nih.gov/assembly/GCA_004568035.1) | A | 10 | Urine | Human | Yes | ExPEC |  |  |
| M16 | [SAMN04122819](http://www.ncbi.nlm.nih.gov/biosample/SAMN04122819) | A | 10 | Blood | Human | Yes | ExPEC |  |  |
| M6 | [SAMN04096288](http://www.ncbi.nlm.nih.gov/biosample/SAMN04096288) | A | 10 | Blood | Human | Yes | ExPEC |  |  |
| 103522 | [GCA_003983445.1](http://www.ncbi.nlm.nih.gov/assembly/GCA_003983445.1) | A | 10 | Stool | Human | Yes | -- |  |  |
| 100516 | [GCA_003984305.1](http://www.ncbi.nlm.nih.gov/assembly/GCA_003984305.1) | A | 10 | Stool | Human | Yes | ETEC |  |  |
| FWSEC0054 | [GCA_005046785.1](http://www.ncbi.nlm.nih.gov/assembly/GCA_005046785.1) | A | 10 | M.D. | Human | M.D. | STEC |  |  |
| G19 | [GCF_001575305.1](http://www.ncbi.nlm.nih.gov/assembly/GCF_001575305.1) | A | 10 | Mastite | Bovine | Yes | ExPEC |  |  |
| 67-965 | [GCA_002243905.1](http://www.ncbi.nlm.nih.gov/assembly/GCA_002243905.1) | A | 10 | Urine | M.D. | M.D. | -- |  |  |
| HVH 209 (4-3062651) | [GCA_000459235.1](http://www.ncbi.nlm.nih.gov/assembly/GCA_000459235.1) | A | 10 | Blood | Human | Yes | ExPEC |  |  |
| Win2013_WWKa_OUT_18 | [GCA_003144805.1](http://www.ncbi.nlm.nih.gov/assembly/GCA_003144805.1) | A | 10 | Wastewater outflow | NA | No | -- |  |  |
| MOD1-EC6587 | [GCA_002469285.1](http://www.ncbi.nlm.nih.gov/assembly/GCA_002469285.1) | A | 10 | Liver | Chicken | M.D. | -- |  |  |
| NCTC9087 | [GCA_900636145.1](http://www.ncbi.nlm.nih.gov/assembly/GCA_900636145.1) | A | 10 | M.D. | M.D. | M.D. | -- |  |  |
| FWSEC0077 | [GCA_005045415.1](http://www.ncbi.nlm.nih.gov/assembly/GCA_005045415.1) | A | 10 | M.D. | Human | M.D. | STEC |  |  |
| MOD1-EC5691 | [GCA_002226695.1](http://www.ncbi.nlm.nih.gov/assembly/GCA_002226695.1) | A | 10 | Milk | Bovine | M.D. | -- |  |  |
| 39_Esco_HA-DE | [GCA_002442335.1](http://www.ncbi.nlm.nih.gov/assembly/GCA_002442335.1) | A | 10 | Rectal swab | Human | No | -- | [29051750](https://www.ncbi.nlm.nih.gov/pubmed/29051750) |  |
| STEC 2708 | [GCF_001607685.1](http://www.ncbi.nlm.nih.gov/assembly/GCF_001607685.1) | A | 10 | Stool | Human | Yes | STEC |  |  |
| MOD1-EC5660 | [GCA_002227365.1](http://www.ncbi.nlm.nih.gov/assembly/GCA_002227365.1) | A | 10 | Stool | Pig | M.D. | -- |  |  |
| 2185 | [GCA_002319385.1](http://www.ncbi.nlm.nih.gov/assembly/GCA_002319385.1) | A | 10 | M.D. | Pig | M.D. | STEC |  |  |
| 2.3916 | [GCA_000194535.2](http://www.ncbi.nlm.nih.gov/assembly/GCA_000194535.2) | A | 10 | M.D. | Pig | M.D. | STEC |  |  |
| VL2874 | [GCF_001441335.1](http://www.ncbi.nlm.nih.gov/assembly/GCF_001441335.1) | A | 10 | Milk | Bovine | Yes | ExPEC |  |  |
| VL2790 | [GCF_001748545.1](http://www.ncbi.nlm.nih.gov/assembly/GCF_001748545.1) | A | 10 | M.D. | Bovine | Yes | ExPEC |  |  |
| VL3009 | [GCF_001748625.1](http://www.ncbi.nlm.nih.gov/assembly/GCF_001748625.1) | A | 10 | M.D. | Bovine | Yes | ExPEC |  |  |
| MOD1-EC5813 | [GCA_002474495.1](http://www.ncbi.nlm.nih.gov/assembly/GCA_002474495.1) | A | 10 | Pleural cavity | Pig | Yes | ExPEC |  |  |
| upec-83 | [GCA_000776355.1](http://www.ncbi.nlm.nih.gov/assembly/GCA_000776355.1) | A | 10 | Urine | Human | Yes | ExPEC | [25373147](https://www.ncbi.nlm.nih.gov/pubmed/25373147) |  |
| MOD1-EC5673 | [GCA_002227115.1](http://www.ncbi.nlm.nih.gov/assembly/GCA_002227115.1) | A | 10 | Stool | Bovine | M.D. | -- |  |  |
| MOD1-EC5677 | [GCA_002226905.1](http://www.ncbi.nlm.nih.gov/assembly/GCA_002226905.1) | A | 10 | Stool | Bovine | M.D. | -- |  |  |
| SC356 | [GCA_003358185.1](http://www.ncbi.nlm.nih.gov/assembly/GCA_003358185.1) | A | 10 | Surface water | NA | No | EPEC |  |  |
| SC352 | [GCA_003358245.1](http://www.ncbi.nlm.nih.gov/assembly/GCA_003358245.1) | A | 10 | Surface water | NA | No | EPEC |  |  |
| 98-3133 (O157:H16) | [GCA_000618005.1](http://www.ncbi.nlm.nih.gov/assembly/GCA_000618005.1) | A | 10 | M.D. | M.D. | M.D. | EPEC | [25103754](https://www.ncbi.nlm.nih.gov/pubmed/25103754) |  |
| TW15901 | [GCA_000304075.1](http://www.ncbi.nlm.nih.gov/assembly/GCA_000304075.1) | A | 10 | Beef | M.D. | No | EPEC |  |  |
| 3.2303 | [GCA_000194645.2](http://www.ncbi.nlm.nih.gov/assembly/GCA_000194645.2) | A | 10 | Water | M.D. | No | EPEC |  |  |
| FWSEC0085 | [GCA_005045345.1](http://www.ncbi.nlm.nih.gov/assembly/GCA_005045345.1) | A | 10 | Stool | Human | M.D. | EPEC |  |  |
| EPEC 2669 | [GCA_001742565.1](http://www.ncbi.nlm.nih.gov/assembly/GCA_001742565.1) | A | 10 | Stool | Human | M.D. | EPEC |  |  |
| EPEC 631 | [GCA_001742485.1](http://www.ncbi.nlm.nih.gov/assembly/GCA_001742485.1) | A | 10 | Stool | Human | M.D. | EPEC |  |  |
| EPEC 536 | [GCA_001742465.1](http://www.ncbi.nlm.nih.gov/assembly/GCA_001742465.1) | A | 10 | Stool | Human | Yes | EPEC |  |  |
| EPEC 720 | [GCA_001742475.1](http://www.ncbi.nlm.nih.gov/assembly/GCA_001742475.1) | A | 10 | Stool | Human | M.D. | EPEC |  |  |
| VREC0305 | [GCA_900479635.1](http://www.ncbi.nlm.nih.gov/assembly/GCA_900479635.1) | A | 10 | Stool | Pig | No | -- |  |  |
| FHI30 | [GCF_000951895.1](http://www.ncbi.nlm.nih.gov/assembly/GCF_000951895.1) | A | 10 | Stool | Human | Yes | STEC |  |  |
| FWSEC0089 | [GCA_005045745.1](http://www.ncbi.nlm.nih.gov/assembly/GCA_005045745.1) | A | 10 | M.D. | M.D. | M.D. | STEC |  |  |
| STEC 2953 | [GCF_001607895.1](http://www.ncbi.nlm.nih.gov/assembly/GCF_001607895.1) | A | 10 | Stool | Human | Yes | STEC |  |  |
| STEC 2236 | [GCF_001607125.1](http://www.ncbi.nlm.nih.gov/assembly/GCF_001607125.1) | A | 10 | Stool | Human | Yes | STEC |  |  |
| fhi | [GCF_000752175.1](http://www.ncbi.nlm.nih.gov/assembly/GCF_000752175.1) | A | 10 | Stool | Human | Yes | STEC |  |  |
| FWSEC0438 | [GCA_005041235.1](http://www.ncbi.nlm.nih.gov/assembly/GCA_005041235.1) | A | 10 | River water | NA | No | STEC |  |  |
| FWSEC0400 | [GCA_005041485.1](http://www.ncbi.nlm.nih.gov/assembly/GCA_005041485.1) | A | 10 | Intake water | NA | No | STEC |  |  |
| FWSEC0049 | [GCA_005046385.1](http://www.ncbi.nlm.nih.gov/assembly/GCA_005046385.1) | A | 10 | Manure | Bovine | No | STEC |  |  |
| FWSEC0625 | [GCA_005037955.1](http://www.ncbi.nlm.nih.gov/assembly/GCA_005037955.1) | A | 10 | M.D. | M.D. | M.D. | STEC |  |  |
| FWSEC0471 | [GCA_005039655.1](http://www.ncbi.nlm.nih.gov/assembly/GCA_005039655.1) | A | 10 | M.D. | Human | M.D. | STEC |  |  |
| FWSEC0050 | [GCA_005046835.1](http://www.ncbi.nlm.nih.gov/assembly/GCA_005046835.1) | A | 10 | Manure | Bovine | No | STEC |  |  |
| NCTC9100 | [GCA_900636235.1](http://www.ncbi.nlm.nih.gov/assembly/GCA_900636235.1) | A | 10 | M.D. | M.D. | M.D. | -- |  |  |
| 400714 aEPEC | [GCF_001285265.1](http://www.ncbi.nlm.nih.gov/assembly/GCF_001285265.1) | A | 10 | Stool | Human | Yes | EPEC | [30127495](https://www.ncbi.nlm.nih.gov/pubmed/30127495) |  |
| C260_92 | [GCA_000446325.1](http://www.ncbi.nlm.nih.gov/assembly/GCA_000446325.1) | A | 10 | Stool | Human | M.D. | EPEC |  |  |
| C213_10 | [GCA_000446225.1](http://www.ncbi.nlm.nih.gov/assembly/GCA_000446225.1) | A | 10 | Stool | Human | M.D. | EPEC |  |  |
| MOD1-ECOR25 | [GCA_003334065.1](http://www.ncbi.nlm.nih.gov/assembly/GCA_003334065.1) | A | 10 | M.D. | Dog | M.D. | EPEC |  |  |
| ECOR25 | [GCA_002190605.1](http://www.ncbi.nlm.nih.gov/assembly/GCA_002190605.1) | A | 10 | M.D. | Dog | M.D. | EPEC |  |  |
| UMEA 3317-1 | [GCA_000461215.1](http://www.ncbi.nlm.nih.gov/assembly/GCA_000461215.1) | A | 10 | Urine | Human | Yes | ExPEC/ EPEC |  | Isolated in 1995, LEE+ ^b^; patient with UTI developed urosepsis. |
| 505393_aEPEC | [GCF_001277455.1](http://www.ncbi.nlm.nih.gov/assembly/GCF_001277455.1) | A | 10 | Stool | Human | No | EPEC | [30127495](https://www.ncbi.nlm.nih.gov/pubmed/30127495) |  |
| 700337 | [GCF_001284305.1](http://www.ncbi.nlm.nih.gov/assembly/GCF_001284305.1) | A | 10 | Stool | M.D. | M.D. | EPEC |  |  |
| 102014_ aEPEC | [GCF_001284445.1](http://www.ncbi.nlm.nih.gov/assembly/GCF_001284445.1) | A | 10 | Stool | Human | No | EPEC |  |  |
| 4581-2 | [GCA_003651325.1](http://www.ncbi.nlm.nih.gov/assembly/GCA_003651325.1) | A | 10 | Stool | Human | Yes | EPEC | [30533858, +1](https://www.ncbi.nlm.nih.gov/pubmed/30533858,31338081) |  |
| 401082 | [GCF_001284905.1](http://www.ncbi.nlm.nih.gov/assembly/GCF_001284905.1) | A | 10 | Stool | Human | No | EPEC |  |  |
| 401174_aEPEC | [GCF_001277755.1](http://www.ncbi.nlm.nih.gov/assembly/GCF_001277755.1) | A | 10 | Stool | Human | No | EPEC |  |  |
| 85B | [GCA_000503415.1](http://www.ncbi.nlm.nih.gov/assembly/GCA_000503415.1) | A | 10 | Retail Meat | Chicken | No | EPEC |  |  |
| ECO1194 (O49:H40) | [ERS357855](http://www.ncbi.nlm.nih.gov/biosample/ERS357855) | A | 10 | Blood | Human | Yes | ExPEC/ EPEC | [28720578](https://www.ncbi.nlm.nih.gov/pubmed/28720578) | Isolated in 2008, LEE+ |
| VREC0450 | [GCA_900479355.1](http://www.ncbi.nlm.nih.gov/assembly/GCA_900479355.1) | A | 10 | Stool | Bovine | No | EPEC |  |  |
| A130 | [GCA_003304335.1](http://www.ncbi.nlm.nih.gov/assembly/GCA_003304335.1) | A | 10 | Stool | Human | No | EPEC |  |  |
| 252 | [GCA_007858995.1](http://www.ncbi.nlm.nih.gov/assembly/GCA_007858995.1) | A | 10 | Urine | Human | Yes | ExPEC/ EPEC | [31649078](https://www.ncbi.nlm.nih.gov/pubmed/31649078) | Isolated in 1999, LEE+, strain classified as EPEC |
| 200781_aEPEC | [GCF_001283165.1](http://www.ncbi.nlm.nih.gov/assembly/GCF_001283165.1) | A | 10 | Stool | Human | No | EPEC | [30127495](https://www.ncbi.nlm.nih.gov/pubmed/30127495) |  |
| 602370_aEPEC | [GCF_001277415.1](http://www.ncbi.nlm.nih.gov/assembly/GCF_001277415.1) | A | 10 | Stool | Human | Yes | EPEC | [30127495](https://www.ncbi.nlm.nih.gov/pubmed/30127495) |  |
| 202973_aEPEC | [GCF_001277735.1](http://www.ncbi.nlm.nih.gov/assembly/GCF_001277735.1) | A | 10 | Stool | Human | Yes | EPEC | [30127495](https://www.ncbi.nlm.nih.gov/pubmed/30127495) |  |
| 200439_aEPEC | [GCF_001285345.1](http://www.ncbi.nlm.nih.gov/assembly/GCF_001285345.1) | A | 10 | Stool | Human | Yes | EPEC | [30127495](https://www.ncbi.nlm.nih.gov/pubmed/30127495) |  |
| 102010_aEPEC | [GCF_001283665.1](http://www.ncbi.nlm.nih.gov/assembly/GCF_001283665.1) | A | 10 | Stool | Human | No | EPEC | [30127495](https://www.ncbi.nlm.nih.gov/pubmed/30127495) |  |
| 202317_aEPEC | [GCF_001285625.1](http://www.ncbi.nlm.nih.gov/assembly/GCF_001285625.1) | A | 10 | Stool | Human | No | EPEC | [30127495](https://www.ncbi.nlm.nih.gov/pubmed/30127495) |  |
| 302700 | [GCF_001283785.1](http://www.ncbi.nlm.nih.gov/assembly/GCF_001283785.1) | A | 10 | Stool | Human | No | EPEC | [30127495](https://www.ncbi.nlm.nih.gov/pubmed/30127495) |  |
| 300814_aEPEC | [GCF_001283945.1](http://www.ncbi.nlm.nih.gov/assembly/GCF_001283945.1) | A | 10 | Stool | Human | No | EPEC | [30127495](https://www.ncbi.nlm.nih.gov/pubmed/30127495) |  |
| 300812 aEPEC | [GCF_001284565.1](http://www.ncbi.nlm.nih.gov/assembly/GCF_001284565.1) | A | 10 | Stool | Human | No | EPEC | [30127495](https://www.ncbi.nlm.nih.gov/pubmed/30127495) |  |

Abbreviations: M.D., missing data; N.A., Not applicable; EPEC, enteropathogenic *Escherichia coli*; ExPEC, extraintestinal pathogenic *E. coli*; ETEC, enterotoxigenic *E. coli*; STEC, Shiga toxin-producing *E. coli*; EHEC, enterohemorrhagic *E. coli*.

^a^ Pathotypes were defined by the presence in their genome of specific virulence factors, as follows: *stx* for STEC; *stx* and LEE for EHEC; *elt* or *est* for ETEC; LEE for EPEC; ExPEC strains were defined by their isolation from confirmed infections in extraintestinal sites; --, unknown pathotype.

^b^ LEE+, strain that carries the locus of enterocyte effacement.

**Table S2.** Resistance related genes found in the UPEC 252 genome

| **Sequence** | **Gene** | **Coverage** | **% Coverage** | **% Identity** | **Accession No** |
| --- | --- | --- | --- | --- | --- |
| NODE_14_length_119506_cov_15.900393 | (Bla)Penicillin_Binding_Protein  *E. coli* | 1-1902/1902 | 100 | 100 | CP002291:664439-666340 |
| NODE_3_length_291521_cov_19.525749 | (Bla)*ampC2_E. coli* | 1-1134/1134 | 100 | 100 | CP002970:332756-333889 |
| NODE_4_length_268620_cov_16.805358 | (Tet)*tet*(34) | 66-421/465 | 76.34 | 75.35 | AB061440:306-770 |
| NODE_4_length_268620_cov_16.805358 | (Bla)*ampH*_*E. coli* | 1-1158/1158 | 100 | 100 | AP012030:395554-396711 |
| NODE_6_length_251389_cov_16.163650 | (Bla)*ampC1_E. coli* | 1-1305/1305 | 100 | 100 | FN649414:2765051-2766355 |
| NODE_11_length_151464_cov_19.824676 | *cpxA* | 1-1374/1374 | 100 | 98.98 | BA000007.3:4905062-4903688 |
| NODE_12_length_148029_cov_19.346615 | *crp* | 1-633/633 | 100 | 99.84 | AP009048.1:4154296-4153663 |
| NODE_13_length_141723_cov_14.239435 | *mdtG* | 661-1227/1227 | 46.21 | 100 | CP000800.1:1192954-1191727 |
| NODE_13_length_141723_cov_14.239435 | *mdtG* | 1-670/1227 | 54.6 | 98.95 | CP000800.1:1192954-1191727 |
| NODE_13_length_141723_cov_14.239435 | *mdtH* | 1-1209/1209 | 100 | 99.92 | U00096:1125326-1124117 |
| NODE_14_length_119506_cov_15.900393 | *kdpE* | 1-678/678 | 100 | 100 | U00096.3:721733-721055 |
| NODE_15_length_112375_cov_16.739746 | *emrB* | 1-1539/1539 | 100 | 100 | U00096:2812615-2814154 |
| NODE_15_length_112375_cov_16.739746 | *emrA* | 1-1173/1173 | 100 | 100 | AP009048:2810082-2811255 |
| NODE_15_length_112375_cov_16.739746 | *emrR* | 1-531/531 | 100 | 100 | U00096.3:2810769-2811300 |
| NODE_1_length_322751_cov_13.573860 | *Klebsiella_pneumoniae_kpnE* | 5-363/363 | 98.62 | 75.83 | AP006725.1:2483889-2484252 |
| NODE_1_length_322751_cov_13.573860 | *Klebsiella_pneumoniae_kpnF* | 6-330/330 | 98.48 | 75.39 | AP006725.1:2484238-2484568 |
| NODE_20_length_87691_cov_14.696428 | *baeR* | 1-723/723 | 100 | 99.86 | AP009048.1:2166412-2167135 |
| NODE_20_length_87691_cov_14.696428 | *baeS* | 1-1404/1404 | 100 | 100 | AP009048:2165012-2166416 |
| NODE_20_length_87691_cov_14.696428 | *mdtC* | 1-3078/3078 | 100 | 98.44 | U00096:2158385-2161463 |
| NODE_20_length_87691_cov_14.696428 | *mdtB* | 1-3123/3123 | 100 | 97.92 | U00096:2155262-2158385 |
| NODE_20_length_87691_cov_14.696428 | *mdtA* | 1-1248/1248 | 100 | 100 | U00096:2154015-2155263 |
| NODE_21_length_77445_cov_16.032670 | *Escherichia_coli_emrE* | 1-333/333 | 100 | 99.1 | Z11877.1:485-818 |
| NODE_22_length_65326_cov_14.334560 | *ugd* | 1-1167/1167 | 100 | 98.54 | U00096:2099613-2098446 |
| NODE_23_length_63932_cov_12.529128 | *marA* | 1-384/384 | 100 | 100 | AP009048.1:1621287-1621671 |
| NODE_28_length_52876_cov_13.795863 | *Escherichia_coli_emrE* | 1-333/333 | 100 | 91.89 | Z11877.1:485-818 |
| NODE_2_length_292435_cov_19.047505 | *acrF* | 1-3105/3105 | 100 | 99.97 | U00096:3415032-3418137 |
| NODE_2_length_292435_cov_19.047505 | *acrE* | 1-1158/1158 | 100 | 100 | U00096:3413863-3415021 |
| NODE_2_length_292435_cov_19.047505 | *acrS* | 1-663/663 | 100 | 100 | U00096:3413465-3412802 |
| NODE_2_length_292435_cov_19.047505 | *bacA* | 1-822/822 | 100 | 100 | U00096.3:3204131-3203309 |
| NODE_2_length_292435_cov_19.047505 | *tolC* | 1-1488/1488 | 100 | 100 | FJ768952:0-1488 |
| NODE_30_length_43053_cov_15.155733 | *mdtK* | 1-1409/1425 | 98.74 | 75.12 | CP014358.1:2162750-2161325 |
| NODE_33_length_39981_cov_19.428740 | *gadX* | 1-825/825 | 100 | 99.88 | AP009048.1:3974604-3975429 |
| NODE_33_length_39981_cov_19.428740 | *gadW* | 1-729/729 | 100 | 94.65 | CP015085.1:2552440-2551711 |
| NODE_33_length_39981_cov_19.428740 | *mdtF* | 1-3114/3114 | 100 | 99.97 | U00096:3660413-3663527 |
| NODE_33_length_39981_cov_19.428740 | *mdtE* | 1-1158/1158 | 100 | 100 | AP009048.1:3981183-3980025 |
| NODE_34_length_39007_cov_18.747891 | *mdtM* | 1-1233/1233 | 100 | 96.35 | U00096.3:4568519-4567286 |
| NODE_3_length_291521_cov_19.525749 | *Escherichia_coli_ampC* | 1-1134/1134 | 100 | 100 | NC_000913.3:4378944-4377810 |
| NODE_3_length_291521_cov_19.525749 | *eptA* | 1-1644/1644 | 100 | 100 | AP009048:4340268-4338624 |
| NODE_3_length_291521_cov_19.525749 | *mdtN* | 1-1032/1032 | 100 | 100 | AP009048.1:4307588-4306556 |
| NODE_3_length_291521_cov_19.525749 | *mdtO* | 1-2052/2052 | 100 | 100 | AP009048.1:4306557-4304505 |
| NODE_3_length_291521_cov_19.525749 | *mdtP* | 1-1467/1467 | 100 | 99.93 | AP009048.1:4304509-4303042 |
| NODE_40_length_19866_cov_20.097067 | Nocardia_rifampin_resistant_beta-subunit_of_RNA_polymerase_(*rpoB2*) | 2980-3332/3489 | 10.03 | 75.56 | AP006618.1:4835199-4838688 |
| NODE_4_length_268620_cov_16.805358 | *Escherichia_coli_ampH* | 1-1158/1158 | 100 | 100 | AP012030.1:396711-395553 |
| NODE_4_length_268620_cov_16.805358 | *acrB* | 1-3150/3150 | 100 | 99.97 | U00096.3:484403-481253 |
| NODE_4_length_268620_cov_16.805358 | *Escherichia_coli_acrA* | 1-1194/1194 | 100 | 100 | U00096.3:485619-484425 |
| NODE_59_length_3766_cov_14.707337 | *Escherichia_coli_emrE* | 1-133/333 | 39.94 | 99.25 | Z11877.1:485-818 |
| NODE_5_length_264695_cov_15.010666 | *Escherichia_coli_mdfA* | 1-1233/1233 | 100 | 98.38 | JQ394987:0-1233 |
| NODE_5_length_264695_cov_15.010666 | *msbA* | 1-1749/1749 | 100 | 100 | U00096.3:966620-968369 |
| NODE_6_length_251389_cov_16.163650 | *mphB* | 1-477/477 | 100 | 98.32 | AE005174.2:3397370-3397847 |
| NODE_6_length_251389_cov_16.163650 | *acrD* | 1-3114/3114 | 100 | 100 | AP009048.1:2586250-2589364 |
| NODE_6_length_251389_cov_16.163650 | *evgS* | 1-3594/3594 | 100 | 100 | U00096:2484373-2487967 |
| NODE_6_length_251389_cov_16.163650 | *evgA* | 1-615/615 | 100 | 100 | BA000007.3:3212025-3212640 |
| NODE_6_length_251389_cov_16.163650 | *emrK* | 1-1056/1056 | 100 | 100 | D78168:536-1592 |
| NODE_6_length_251389_cov_16.163650 | *emrY* | 1-1539/1539 | 100 | 99.94 | D78168:1591-3130 |
| NODE_75_length_1167_cov_12.041346 | *Escherichia_coli_emrE* | 89-333/333 | 73.57 | 98.78 | Z11877.1:485-818 |
| NODE_7_length_237541_cov_15.161296 | *yojI* | 1-1644/1644 | 100 | 100 | U00096.3:2308615-2306971 |
| NODE_7_length_237541_cov_15.161296 | *pmrF* | 1-969/969 | 100 | 100 | U00096:2367070-2368039 |
| NODE_8_length_221796_cov_13.359274 | *hns* | 1-414/414 | 100 | 99.28 | BA000007.3:1738104-1737690 |
| NODE_5_length_264695_cov_15.010666 | *mdf*(A)_1 | 1-1233/1233 | 100 | 99.92 | Y08743 |

**Table S3.** Antimicrobial susceptibility profile of UPEC 252 ^a^

| **Antimicrobial** | **UPEC 252**  **halo (mm)** | **Report** |
| --- | --- | --- |
| amoxicillin-clavulanic acid (20/ 10 μg) | 25 | S |
| aztreonam (30 μg) | 30 | S |
| cefoxitin (30 μg) | 29 | S |
| cefepime (30 μg) | 31 | S |
| ceftazidime (10 μg) | 27 | S |
| ceftriaxone (30 μg) | 29 | S |
| meropenem (10 μg) | 31 | S |
| imipenem (10 μg) | 30 | S |
| ertapenem (10 μg) | 27 | S |
| gentamicin (10 μg) | 19 | S |
| amikacin (30 μg) | 20 | S |
| tigecycline (15 μg) | 22 | S |
| ciprofloxacin (5 μg) | 35 | S |

^a^ Antimicrobial susceptibility test was performed using the disk diffusion

methodology as recommended by BrCAST guideline. The *E. coli* ATCC25922

was used as a quality standard in the assay.

S- Susceptible.


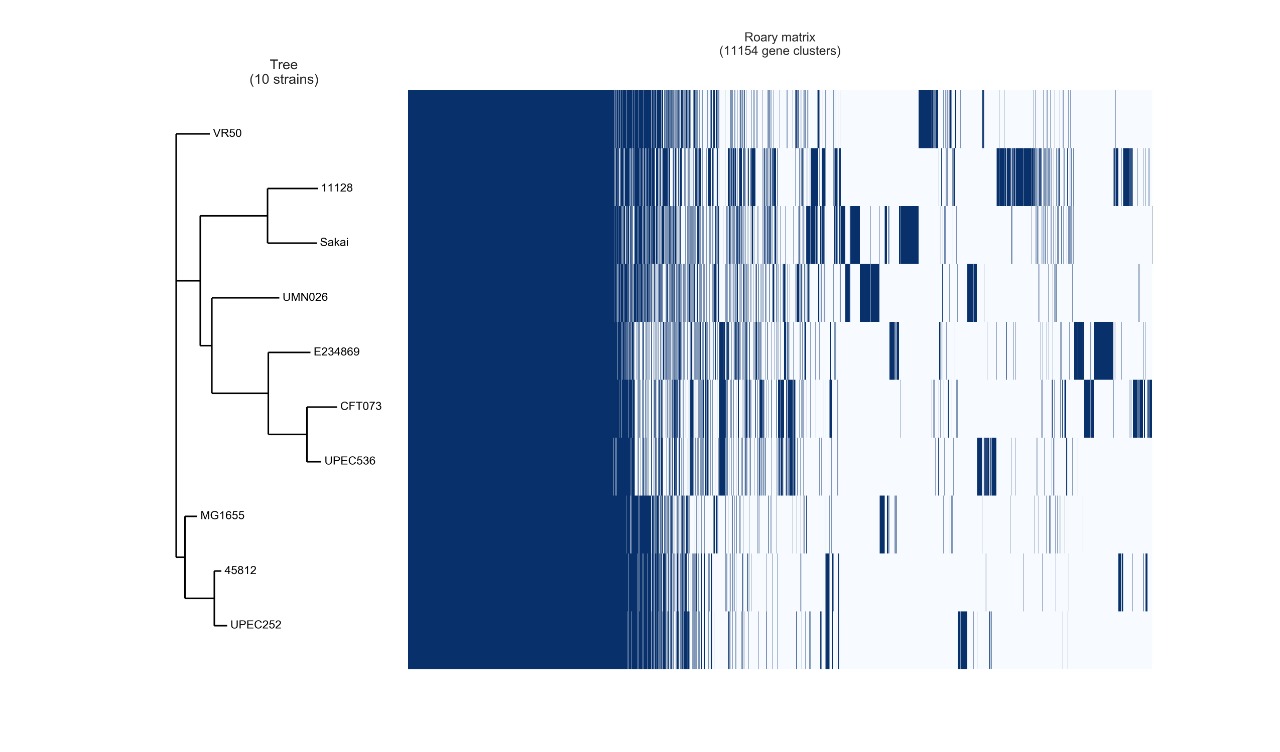


**Figure S3.** **Roary similarity tree and genes matrix.** The similarity tree was built using the pangenome of 10 *E. coli* strains (UPEC 252, 4581-2, 536, CFT073, UMN026, VR50, MG1655, E2348/69, 11128, and Sakai). In the Roary matrix, the blue stripes represent the presence of the gene clusters that comprise the genomes. White stripes represent the absence of the genes.


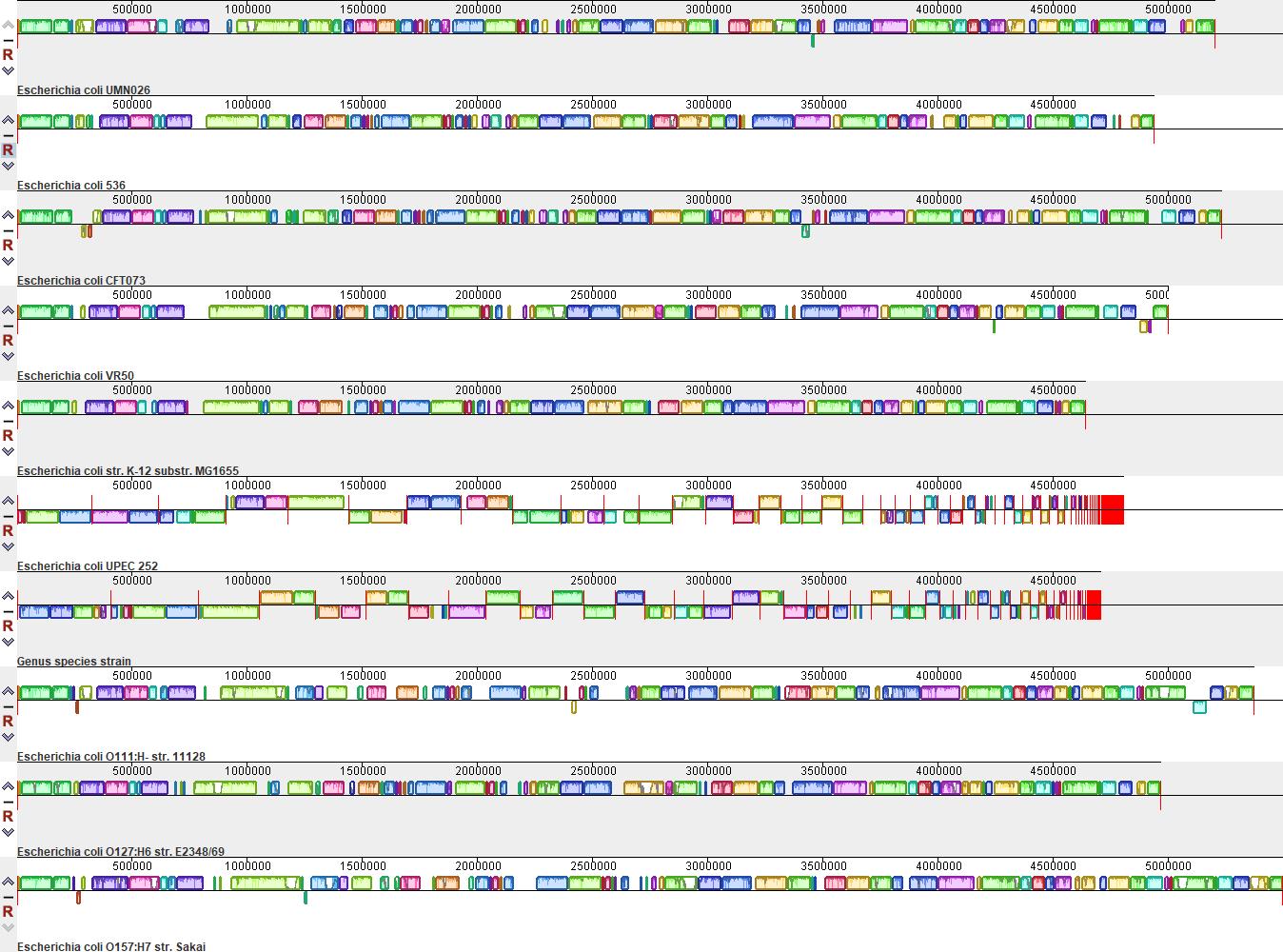


**Figure S4.** **Alignment of UPEC 252 and other nine *E. coli* strains** **(4581-2, 536, CFT073, UMN026, VR50, MG1655,**

**E2348/60, 11128, and Sakai).** The continuous homology blocks are represented by the same color in each genome.


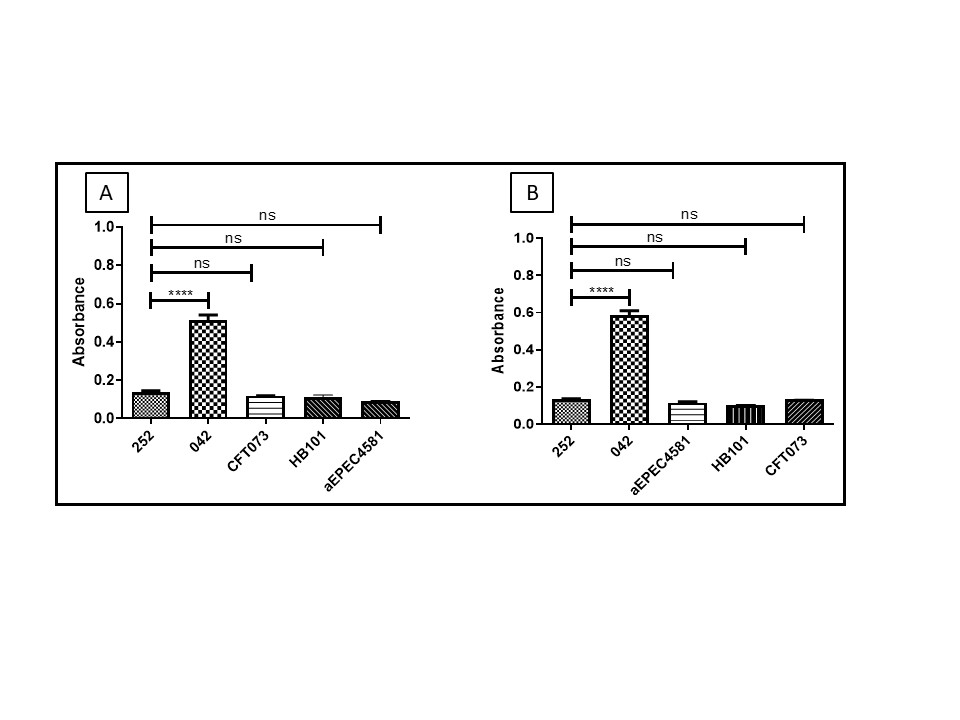


**Figure S5.** **UPEC 252 biofilm formation with incubation periods of 24 (A) and 48 (B) hours, in DMEM + GlutaMAX medium.** Positive control: EAEC prototype strain 042. Negative control: *E. coli* HB101.The One-way ANOVA followed by post hoc Tukey HSD test was used to compare the results. *P-*value, ns P > 0.05, **** *P* ≤ 0.0001.


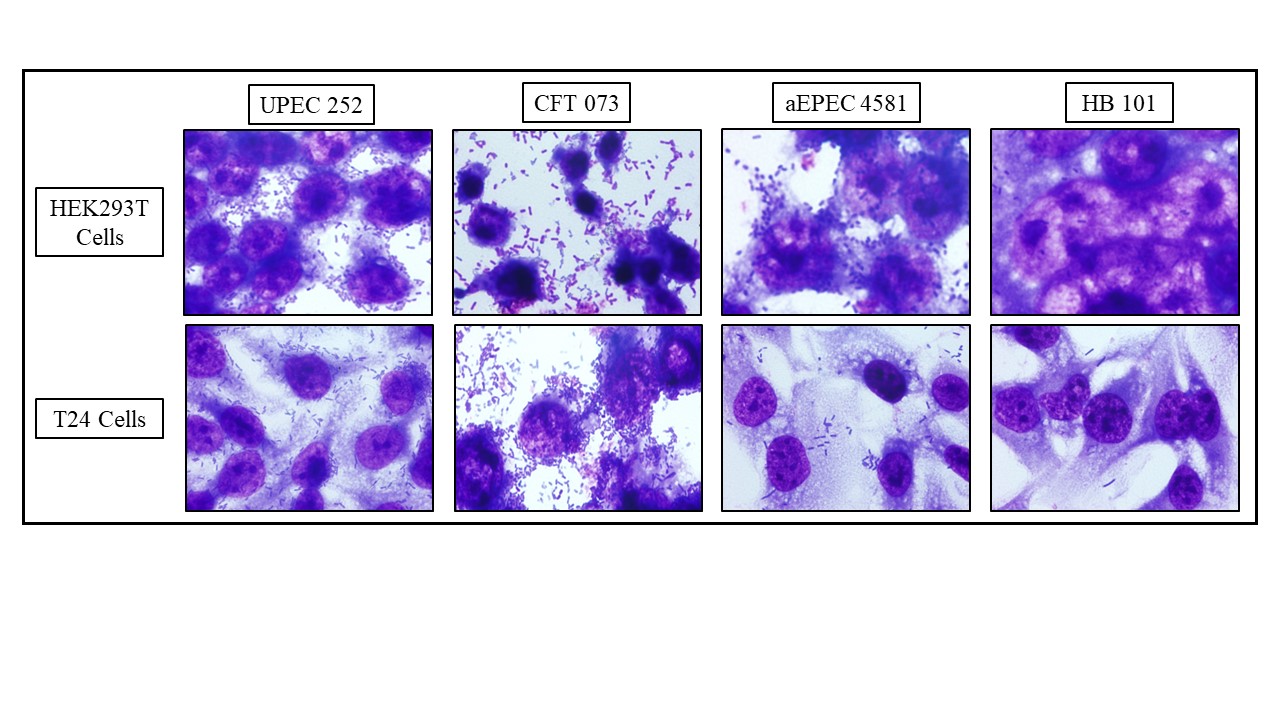


**Figure S6. UPEC 252 interaction assay with urinary tract cell lineages.**Qualitative interaction assay performed with a 3-hour incubation period on

the urinary cell lineages (T24 and HEK 293T), in the absence of D-mannose. Controls: UPEC CFT073; aEPEC 4581-2; non-adherent *E. coli* HB101.


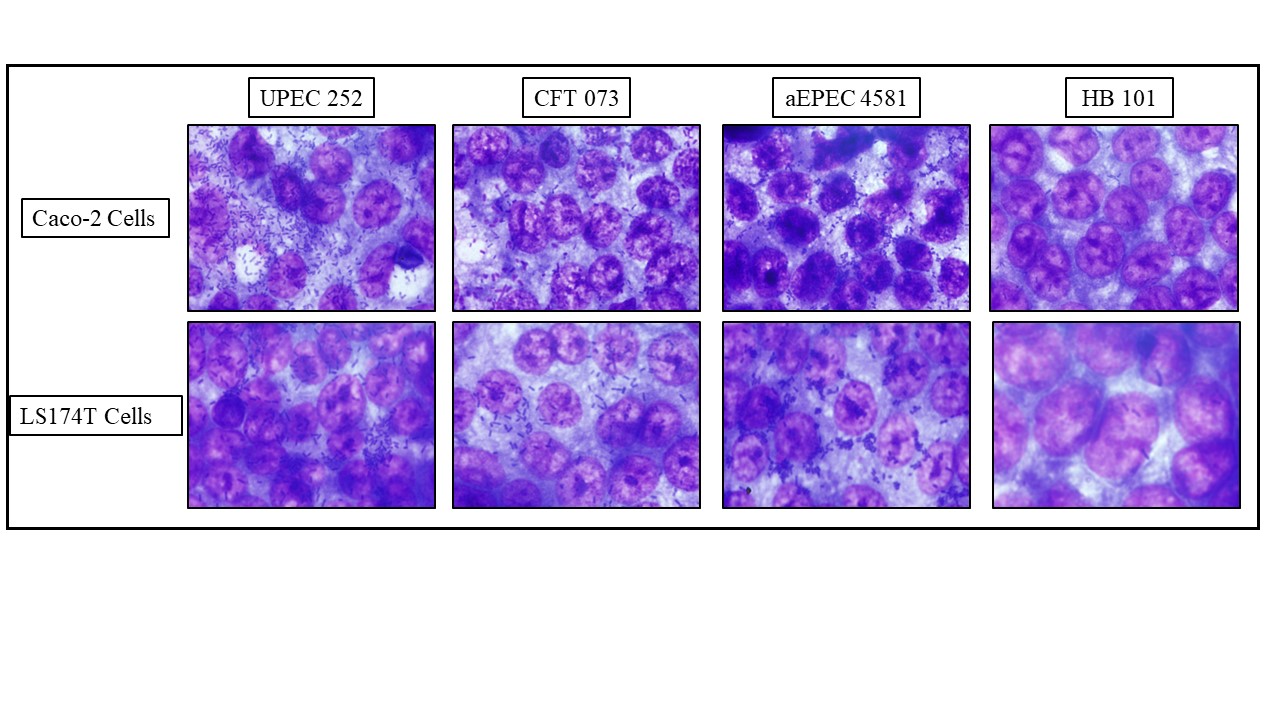


**Figure S7. UPEC 252 interaction assay with intestinal tract cell lineages.**Qualitative interaction assay performed with a 3- hour incubation period in the intestinal tract cells (Caco-2 and LS174T), in the absence of D-mannose. Controls: UPEC CFT073; aEPEC 4581-2; non-adherent *E.* *coli* HB101.

**Figure S8. Quantitative adherence of UPEC 252 in different eukaryotic cell lineages.** Quantitative assays were performed in a 3-hour incubation period, in urinary (T24 and HEK 293T), and intestinal (Caco-2 and LS174T) tract cell lineages, in the absence of D-mannose. The One-way ANOVA followed by *post hoc* Tukey HSD test was used to compare the results obtained in this experiment. *P-*value: *** P ≤ 0.001.


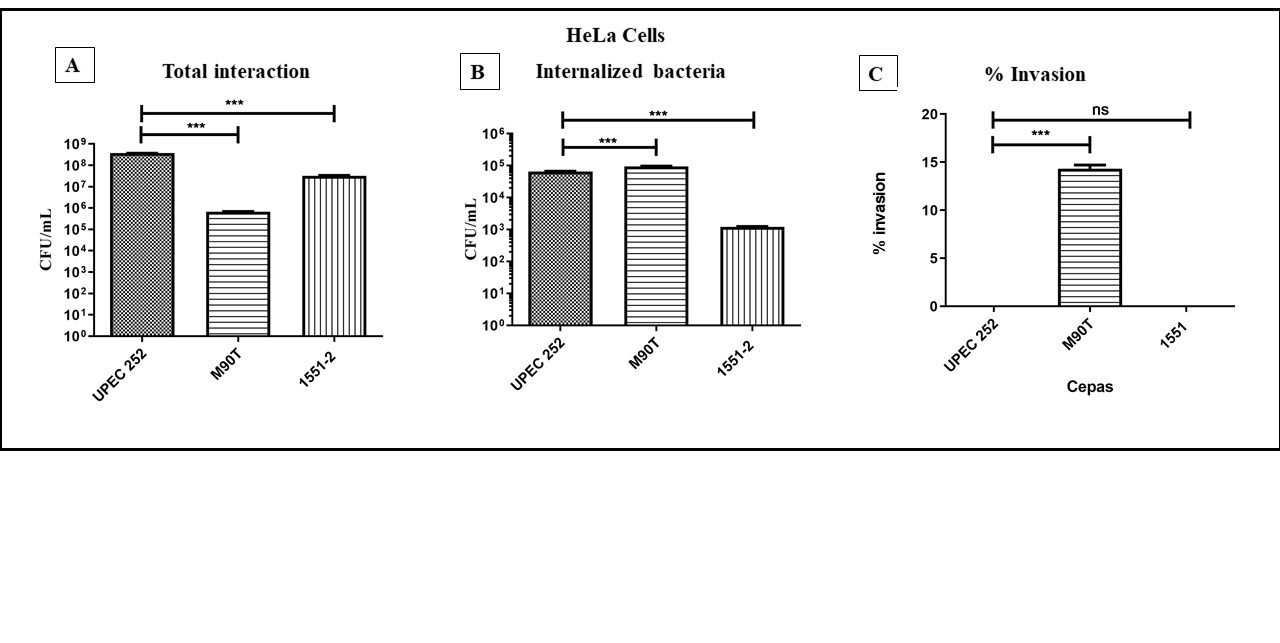


**Figure S9.** **Interaction and invasion ability of UPEC 252 in HeLa cells.** (A) Total cell-associated bacteria recovered after 3 hours of contact. The interaction of UPEC 252 was significantly more efficient than the invasive control strain M90T. (B) Quantitative invasion assay in HeLa cells performed using an infection period of 3 hours, followed by treatment with gentamicin for 1 h to kill extracellular bacteria: number of internalized bacteria after treatment. Although the number of internalized UPEC 252 bacteria was significantly lower than the positive control strain, it was significantly higher than the non-invasive control strain. (C) The invasion index is indicated as the ratio between internalized and total cell-associated bacteria expressed as a percentage. The One-way ANOVA followed by *post hoc* Tukey HSD test was used to compare the results obtained in this experiment. *P*-value: *** *P* <0.001; ns, statistical difference is non-significant (*P* >0.05). Positive control: *Shigella flexneri* M90T. *E. albertii* 1551-2Δ*eae* was used as an adherent non-invasive control. The assays were performed in biological and experimental triplicates. The data presented consist of the mean ± Standard Deviation.


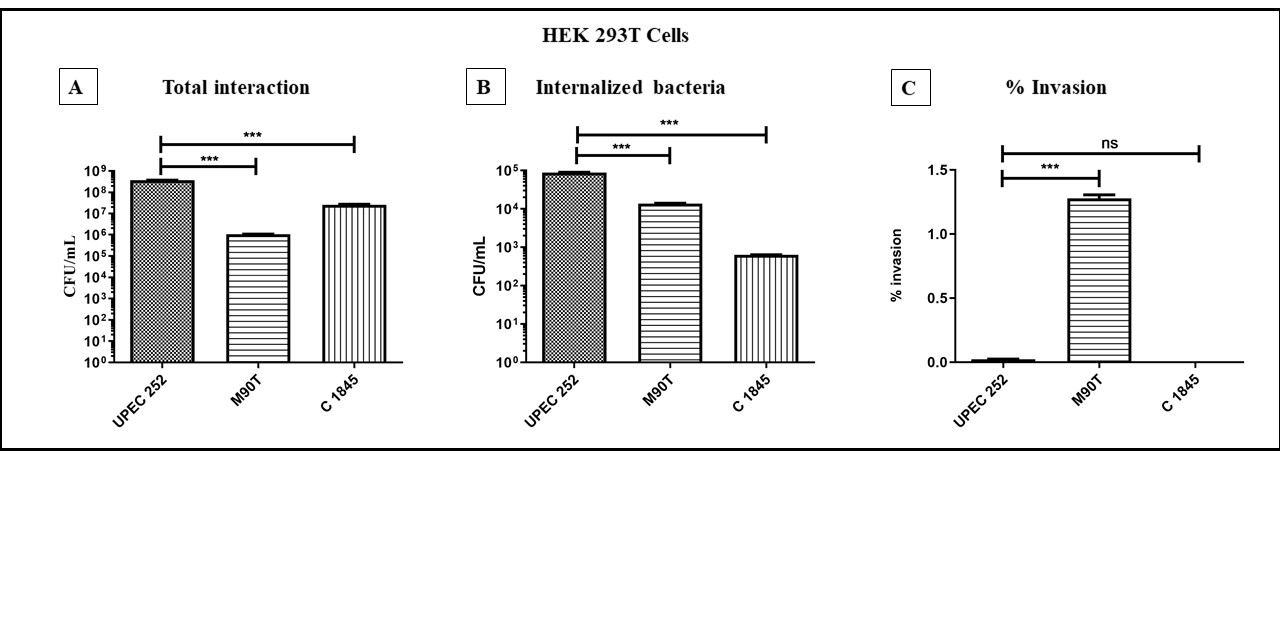


**Figure S10.** **Interaction and invasion ability of UPEC 252 in HEK 293T cells.** (A) Total cell-associated bacteria recovered after 3 hours of contact. The interaction of UPEC 252 was significantly more efficient than the invasive control strain M90T. (B) Quantitative invasion assay in HeLa cells performed using an infection period of 3 hours, followed by treatment with gentamicin for 1 h to kill extracellular bacteria: number of internalized bacteria after treatment. The number of internalized UPEC 252 bacteria was significantly higher than both the positive and non-invasive control strains. (C) The invasion index is indicated as the ratio between internalized and total cell-associated bacteria expressed as a percentage. UPEC 252 was significantly less invasive than the invasive control strain. The One-way ANOVA followed by *post hoc* Tukey HSD test was used to compare the results obtained in this experiment. *P*-value: *** *P* <0.001; ns, statistical difference is non-significant. Positive control: *Shigella flexneri* M90T. *E. coli* C1845 was used as an adherent non-invasive control. The assays were performed in biological and experimental triplicates. The data presented consist of the mean ± Standard Deviation.


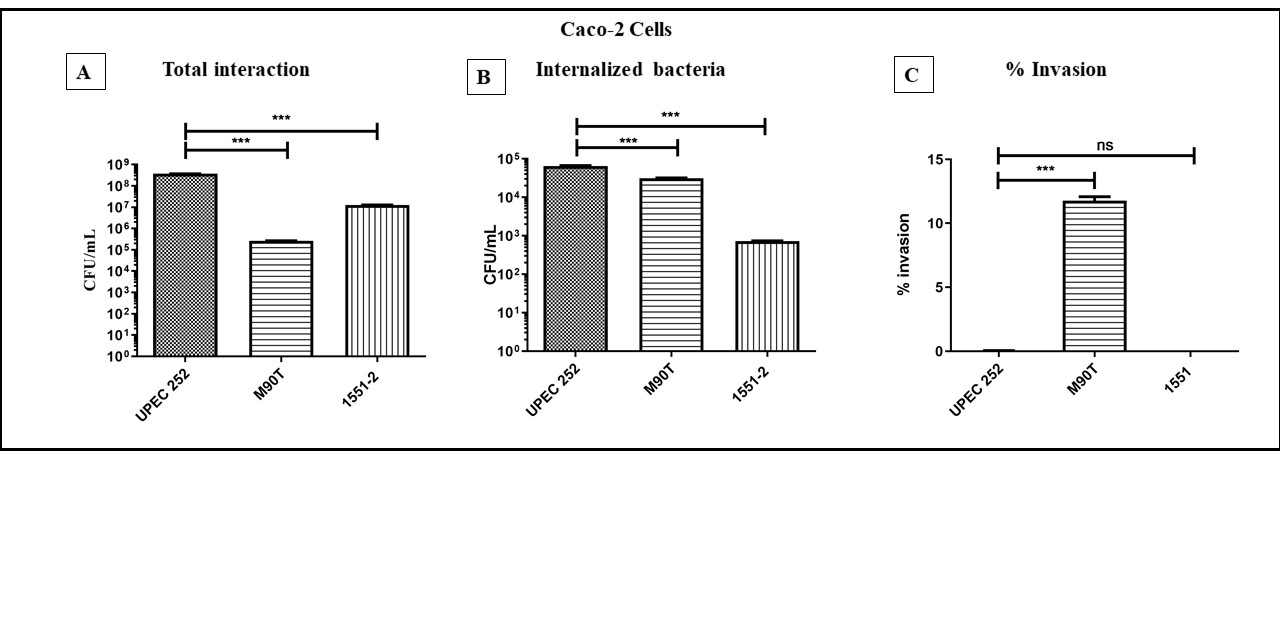


**Figure S11.** **Interaction and invasion ability of UPEC 252 in Caco-2 cells.** (A) Total cell-associated bacteria recovered after 3 hours of interaction. The interaction of UPEC 252 was significantly more efficient than the invasive control strain M90T. (B) Quantitative invasion assay in HeLa cells performed using an infection period of 3 hours, followed by treatment with gentamicin for 1 h to kill extracellular bacteria: number of internalized bacteria after treatment. The number of internalized UPEC 252 bacteria was significantly higher than both the positive and non-invasive control strains. (C) The invasion index is indicated as the ratio between internalized and total cell-associated bacteria expressed as a percentage. UPEC 252 was significantly less invasive than the invasive control strain. The One-way ANOVA followed by post hoc Tukey HSD test was used to compare the results obtained in this experiment. *P-*value: *** *P* <0.001; ns, statistical difference is non-significant. Positive control: *Shigella flexneri* M90T. *E. albertii* 1551-2Δ*eae* was used as an adherent non-invasive control. The assays were performed in biological and experimental triplicates. The data presented consists of the mean ± Standard Deviation.
